# Supplementary material for: COX2 regulates senescence secretome composition and senescence surveillance through PGE2
Source: Cell Rep. 2021 Mar 16;34(11):108860. doi: 10.1016/j.celrep.2021.108860 (PMC7972992; doi:10.1016/j.celrep.2021.108860)
Supplement: Document S1. Figures S1–S5 [file mmc1.pdf]

Cell Reports, Volume *34*

Supplemental information

COX2 regulates senescence secretome composition and senescence surveillance through PGE_2

Susana Gonçalves, Kelvin Yin, Yoko Ito, Adelyne Chan, Ioana Olan, Sarah Gough, Liam Cassidy, Eva Serrao, Stephen Smith, Andrew Young, Masashi Narita, and Matthew Hoare

Supplementary Figure 1

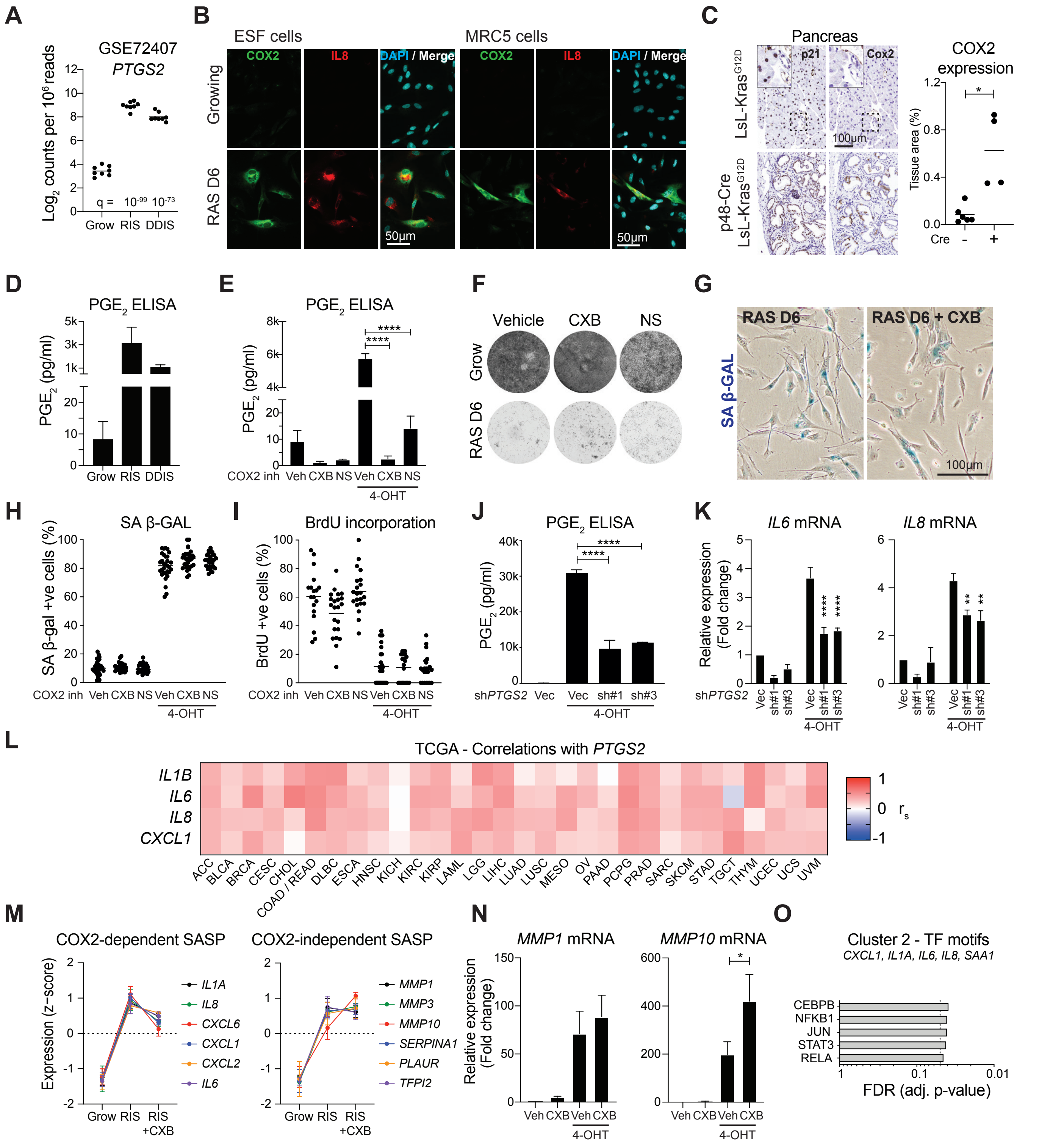

Supplementary figure 1. COX2 is upregulated in senescence and regulates SASP composition, related to Figure 1. (A) Expression of *PTGS2*, the gene that encodes COX2, from previously generated mRNA-Seq (GSE72407) data in growing, RAS-induced senescent (RIS) and DNA-damage-induced senescent (DDIS) ER:HRAS^{G12V} IMR90 cells. (B) Representative immunofluorescence of COX2 and IL8 in growing or RIS ER:HRAS^{G12V} ESF (left) or ER:HRAS^{G12V} MRC5 (right) HDFs cells (scale bar 50μm). (C) Left: representative photomicrographs of immunohistochemistry for p21 (left) and COX2 (right) of pancreatic tissue from p48-cre; LSL-Kras^{G12D} (n = 4) and matched control mice (n = 6); insets demonstrate the staining patterns within normal pancreatic ducts in the control mice (upper) and pancreatic intraepithelial neoplasia (PanIN) in the p48-cre; LSL-Kras^{G12D} mice (lower). Scale bar 100 μm. Right: quantification of Cox2-positive tissue area; bar represents the mean; * P < 0.05; statistical analysis by unpaired t-test with Welch's correction. (D) Prostaglandin E2 (PGE_2) levels by ELISA in growing, RIS or DDIS ER:HRAS^{G12V} IMR90 cells (n = 3 biologically independent experiments). (E) Prostaglandin E2 (PGE_2) levels by ELISA in growing or D6 RIS ER:HRAS^{G12V} IMR90 cells treated with vehicle (Veh), Celecoxib (40μM, CXB) or NS398 (10μM, NS); n = 3 biologically independent experiments; values are mean ± SEM; **** P ≤ 0.0001; statistical analysis by 1-way ANOVA with Sidak's multiple comparisons test. (F) Growing or RIS ER:HRAS^{G12V} IMR90 cells treated with vehicle (Veh), Celecoxib (40μM, CXB) or NS398 (10μM, NS) were analysed for colony forming capacity. (G, H & I) Growing or RIS ER:HRAS^{G12V} IMR90 cells treated with vehicle, CXB or NS were analysed for SA-β GAL expression (G, H) or BrdU incorporation (I) at D6 RAS. Example photomicrographs in (G) from indicated conditions (scale bar 100μm) and summary data in (H); n ≥ 18 biologically independent experiments; bar represents the mean. (J) PGE_2 levels by ELISA in growing or D6 RIS ER:HRAS^{G12V} IMR90 cells expressing shRNAs against *PTGS2* or vector control; n = 3 biologically independent experiments; values are mean ± SEM; **** P ≤ 0.0001; statistical analysis by 1-way ANOVA with Sidak's multiple comparisons test. (K) Growing or RIS ER:HRAS^{G12V} IMR90 cells, expressing shRNAs against *PTGS2* or vector control, were analysed for expression of indicated genes by qRT-PCR; n ≥ 3 biologically independent samples per condition; values are mean ± SEM; **** P ≤ 0.0001 versus RIS / vector condition; statistical analysis by 2-way ANOVA with Tukey's multiple comparisons test; values are mean ± SEM; ** P ≤ 0.01, ****P ≤ 0.0001. (L) Heatmap of correlation coefficients between expression of *PTGS2* and selected cytokines in a pan-cancer analysis of TCGA transcriptomic data; statistical analysis by Spearman's rank correlation (rs). (M) z-score normalised expression from mRNA-Seq of selected canonical SASP components, demonstrating COX2 dependent (left) and COX2-independent (right) genes. (N) Expression of *MMP1* and *MMP10* in growing or RIS ER:HRAS^{G12V} IMR90 cells treated with vehicle or CXB was analysed by qRT-PCR (n = 5 biologically independent replicates for all conditions; values are mean ± SEM; * P ≤ 0.05; statistical analysis by 1-way ANOVA with Sidak's multiple comparisons test. (O) Transcription factor motif enrichment analysis from cluster 2 genes, using TRRUST v2 database.

Supplementary figure 2

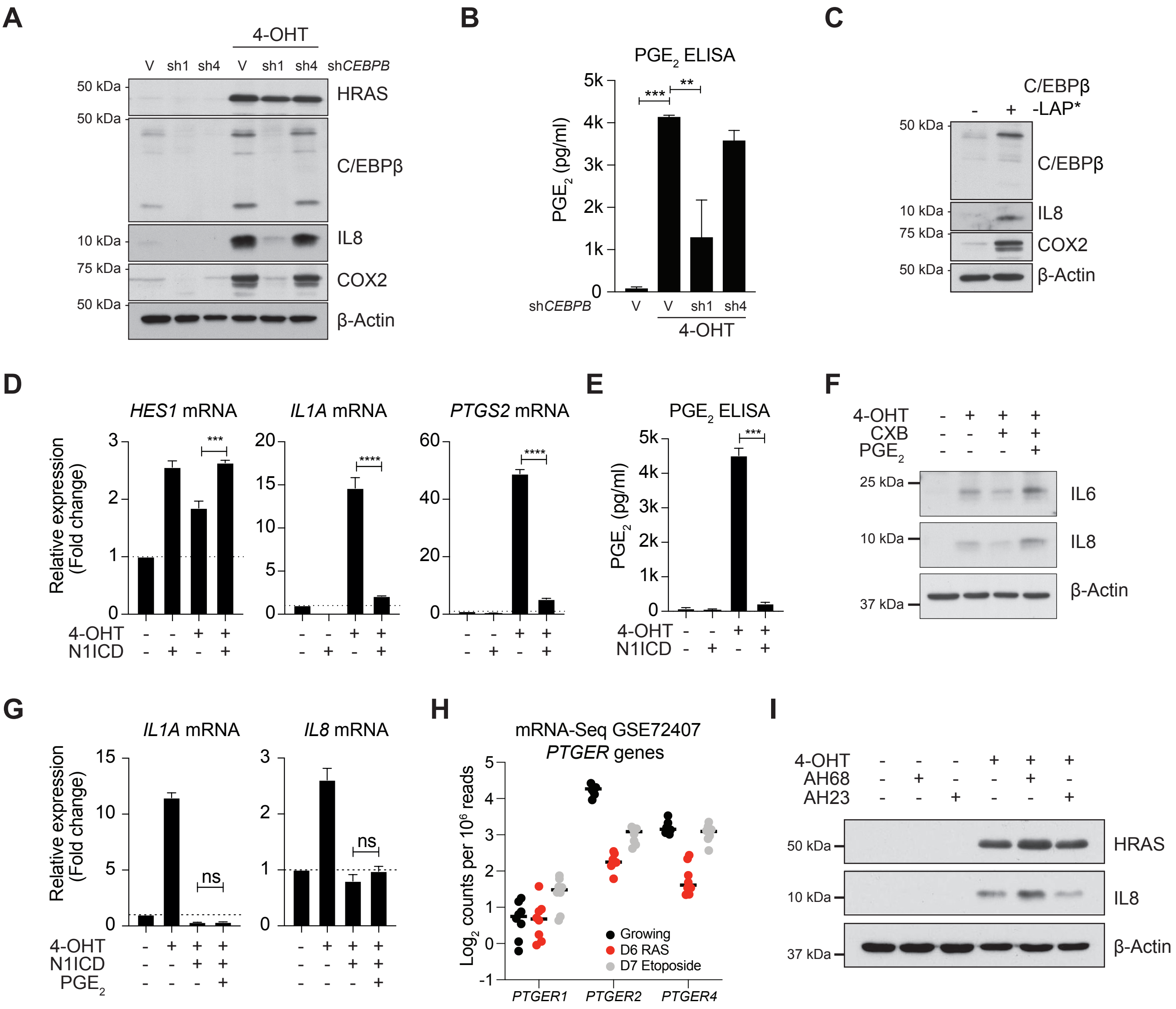

Supplementary figure 2. Upstream regulation of COX2 and downstream regulation of the SASP through PGE_2, related to Figure 2. (A) Growing or RIS ER:HRAS^{G12V} IMR90 cells, expressing shRNAs against *CEBPB* or vector control, were analysed for expression of indicated proteins by immunoblotting (A) and Prostaglandin E2 (PGE_2) levels by ELISA (B); n = 3 biologically independent experiments; values are mean ± SEM; ** P ≤ 0.01, *** P ≤ 0.001; statistical analysis by 1-way ANOVA with Sidak's multiple comparisons test. (C) IMR90 cells expressing the transcriptionally active LAP* form of C/EBPβ or matched vector control were analysed for the indicated proteins by immunoblotting. (D) Expression of *HES1, IL1A* and *COX2* in growing or RIS ER:HRAS^{G12V} IMR90 cells transfected with a transcriptional active NOTCH1-ICD (N1ICD) or vector control were analysed by qRT-PCR; (E) expression of PGE_2 was analysed in the same cells by ELISA; n = 3 biologically independent replicates for all conditions; values are mean ± SEM; statistical analysis by 1-way ANOVA with Sidak's multiple comparisons test; *** P ≤ 0.001, **** P ≤ 0.0001. (F) Expression of IL6 and IL8 in growing or RIS ER:HRAS^{G12V} IMR90 cells treated with vehicle or CXB and vehicle or PGE_2 (10µM) by immunoblotting. (G) Expression of *IL1A* and *IL8* in growing or RIS ER:HRAS^{G12V} IMR90 cells, expressing N1ICD or vector control and treated with vehicle or PGE_2 were analysed by qRT-PCR; n = 3 biologically independent replicates for all conditions; values are mean ± SEM; statistical analysis by 1-way ANOVA with Sidak's multiple comparisons test. (H) Expression of *PTGER* genes, encoding the EP receptors, from previously generated mRNA-Seq (GSE72407) data in growing, RAS-induced senescent (RIS) and DNA-damage-induced senescent (DDIS) ER:HRAS^{G12V} IMR90 cells. *PTGER3* is not detectable in IMR90 cells. (I) Growing or RIS ER:HRAS^{G12V} IMR90 cells, treated with vehicle, AH6809 (10µM) or AH23848 (10µM) were analysed for expression of indicated proteins by immunoblotting.

Supplementary figure 3

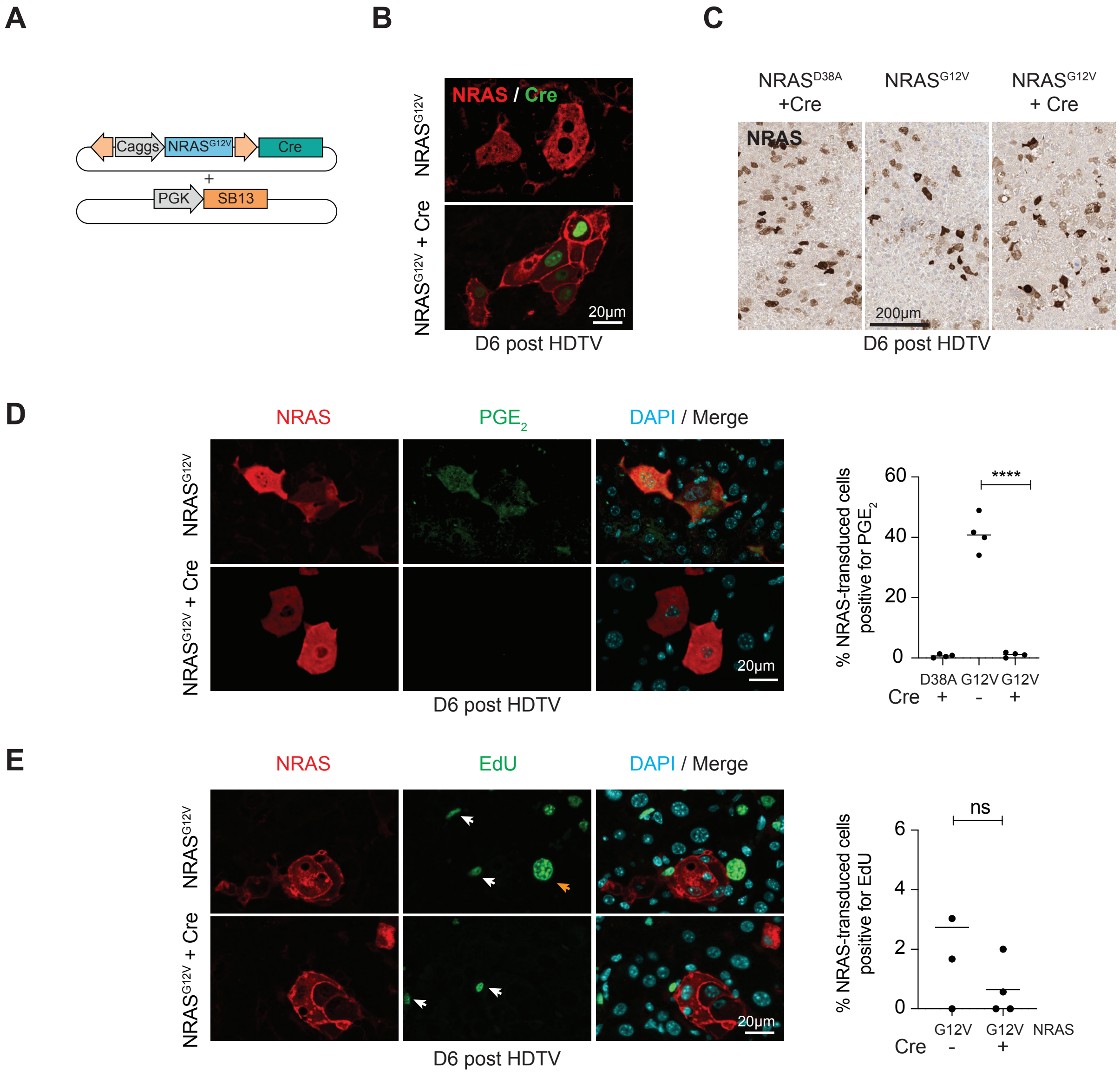

Supplementary figure 3. Cox2 regulates senescence surveillance and tumour suppression *in vivo*, related to Figure 3. (A) Cartoon schematic of the transposon and transposase-containing vectors that were used in the hydrodynamic tail-vein (HDTV) injection experiments. The Cre-recombinase is outside the transposon inverted repeat elements and so is only expressed episomally and transiently after injection. (B) Representative immunofluorescence images of NRAS and Cre in mouse livers at D6 post HDTV injection of the indicated constructs; scale bar 20µm (C) Illustrative photomicrographs of NRAS immunohistochemistry in indicated conditions at D6 post HDTV injection; scale bar 200µm. (D) Representative immunofluorescence images (left) of NRAS and PGE_2 in mouse livers at D6 post HDTV injection of the indicated constructs; scale bar 20µm and quantification of the number of NRAS+ hepatocytes that were also positive for PGE_2 staining (right) in the indicated conditions; dots are individual mice; bars are means; data analysed by 1-way ANOVA with Sidak's multiple comparisons test; ****$P \leq 0.0001$. (E) Representative immunofluorescence images (left) of NRAS and EdU in mouse livers at D6 post HDTV injection of the indicated constructs; note the EdU incorporation in immune cells (white arrowheads) and non-transduced hepatocyte (orange arrowhead); scale bar 20µm; quantification of the number of NRAS+ hepatocytes that were also positive for EdU staining (right) in the indicated conditions; dots are individual mice; bars are means; data analysed by unpaired student's t-test.

Supplementary figure 4

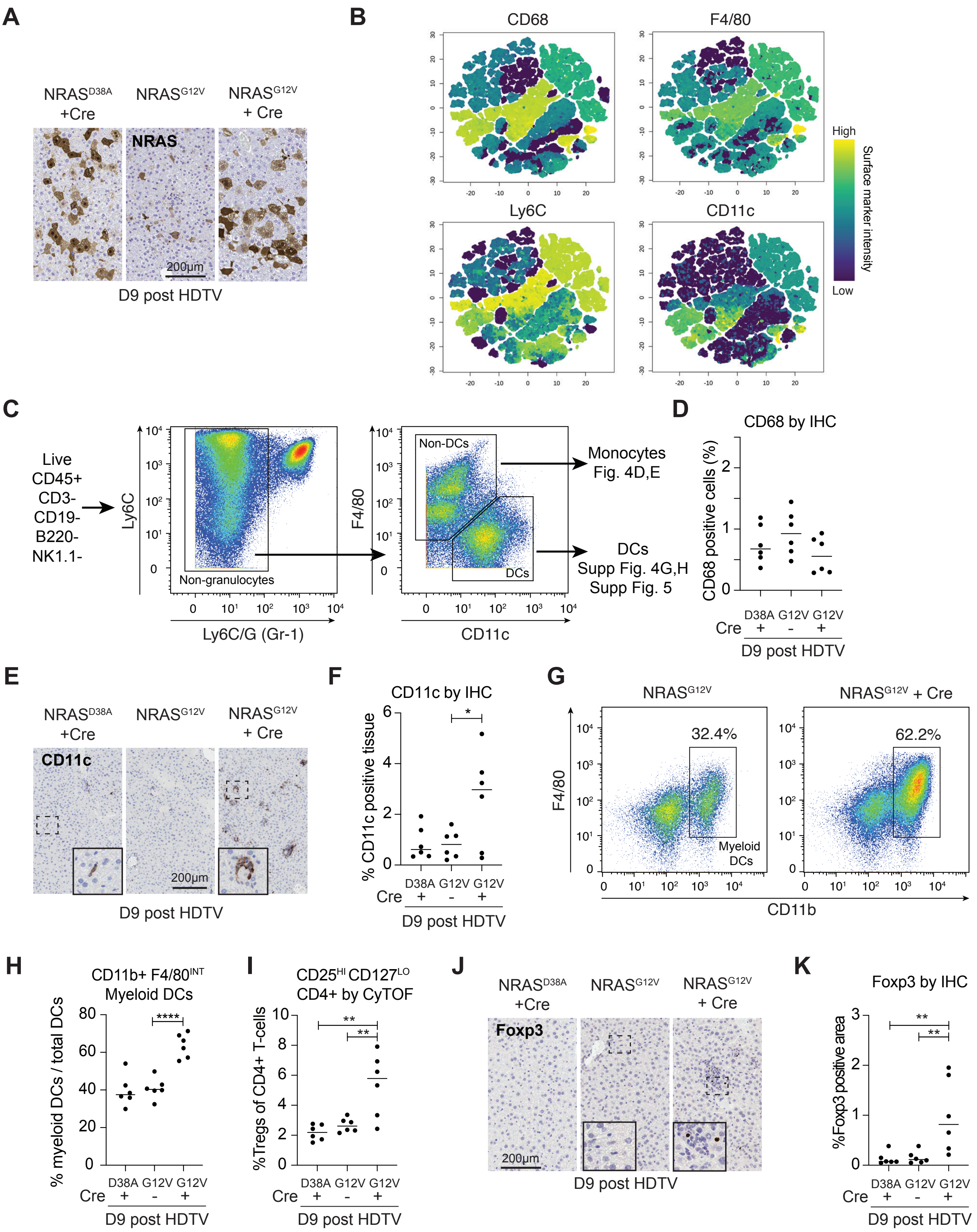

Supplementary figure 4. Cox2 controls the intrahepatic immune microenvironment during senescence surveillance, related to Figure 4. (A) Illustrative photomicrographs of NRAS IHC in indicated conditions at D9 post HDTV injection; scale bar 200µm. (B) t-SNE plots showing hyperspheres of immune cell phenotypes (see Fig. 4 B&C), coloured by median intensity of the indicated markers. The colour range is bounded by the 1st and 99th percentiles of the intensities across all cells. (C) Gating strategy to study a non-granulocytic / non-dendritic cell (DC) myeloid population in figures 4 D&E. (D) Expression of CD68 in the liver by IHC in same mice used for mass-cytometry; dots are individual mice; bars are means. (E) Illustrative photomicrographs of CD11c IHC in indicated conditions at D9 post HDTV injection of the indicated constructs; scale bar 200µm. Insets are higher magnification views of areas in the dotted dashed boxes showing CD11c+ cells. (F) Quantification of CD11c-positive area per liver section, by IHC. Dots are individual mice; bars are means; data analysed by 1-way ANOVA with Sidak's multiple comparisons test; * $P \leq 0.05$. (G, H) Live lineage-negative, dendritic cells (DC) were gated as in supplementary fig. 4C, before analysis of different DC populations based on CD11b and F4/80 expression, identifying myeloid DCs as CD11b+ and F4/80^{INT}; example dot-plots of DC populations from NRAS^{G12V} and NRAS^{G12V}+Cre injected mice are shown in (G) with quantification in (H); dots are individual mice; bars are means; data analysed by 1-way ANOVA with Sidak's multiple comparisons test; **** $P \leq 0.0001$. (I) Relative abundance of CD4+ Tregs across the indicated conditions at D9 post HDTV injection by mass cytometry. Tregs were defined by the surface expression profile CD3+CD4+CD25^{HI}CD127^{LO} and expressed as a proportion of total CD3+CD4+ cells; dots are individual mice; bars are means; data analysed by 1-way ANOVA with Sidak's multiple comparisons test; ** $P \leq 0.01$. (J) Illustrative photomicrographs of Foxp3 IHC in indicated conditions at D9 post HDTV injection of the indicated constructs; scale bar 200µm. Insets are higher magnification views of areas in the dotted dashed boxes showing Foxp3+ cells. (K) Quantification of Foxp3-positive area per liver section, by IHC. Dots are individual mice; bars are means; data analysed by 1-way ANOVA with Sidak's multiple comparisons test; ** $P \leq 0.01$.

Supplementary figure 5

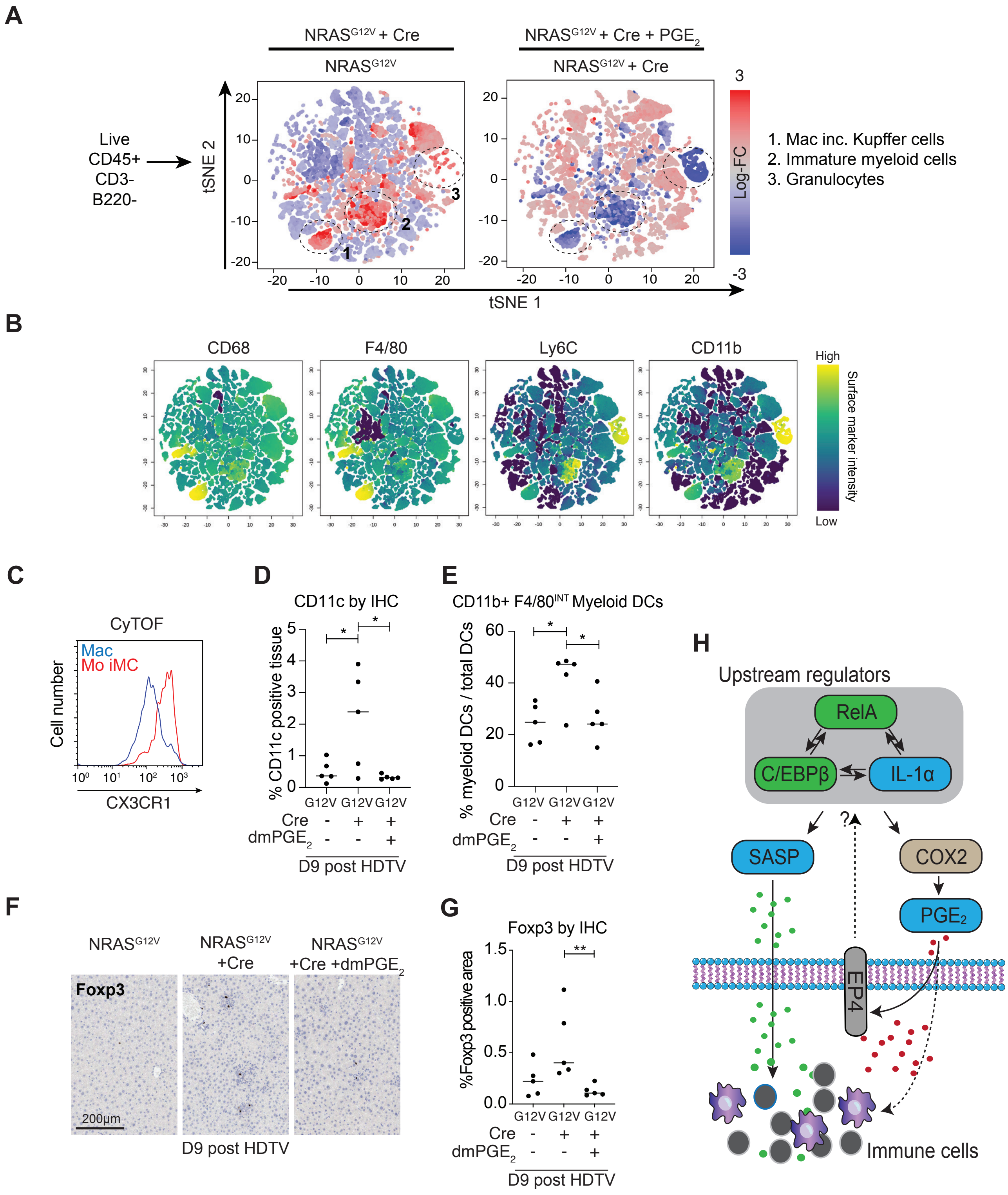

Supplementary figure 5. PGE$_2$ partially rescues Cox2-dependent immune-mediated senescence surveillance, related to Figure 5. (A) t-SNE plots of multiplexed intrahepatic immune cell mass-cytometry data from 15 mice (same conditions as Fig. 5 A-C, n = 5 per condition), using 16 metal-tagged antibodies against immunocyte surface markers (see supplementary table 2). t-SNE plots demonstrate differential abundance of CD45+, non-lymphoid cells from NRAS^{G12V} vs NRAS^{G12V}+Cre (left) and NRAS^{G12V}+Cre vs NRAS^{G12V}+Cre+dmPGE$_2$ (right) mice. Hyperspheres of immune cell phenotypes were identified by typical surface marker expression and coloured according to log-fold change in abundance between conditions. (B) t-SNE plots showing hyperspheres of immune cell phenotypes, coloured by median intensity of the indicated markers. The colour range is bounded by the 1st and 99th percentiles of the intensities across all cells. (C) Histogram demonstrating expression of CX3CR1 on iMCs (CD68^{Intermediate} / F4/80^{Low}) and macrophages (CD68^{High} / F4/80^{High}) from NRAS^{G12V}+Cre injected mouse liver. (D) Quantification of CD11c-positive area per liver section, by IHC in the indicated conditions. Dots are individual mice; bars are means; data analysed by 1-way ANOVA with Sidak's multiple comparisons test; * $P \leq 0.05$. (E) Relative abundance of myeloid DCs across the indicated conditions at D9 post HDTV injection by mass cytometry; see Supp fig 4c for gating strategy. Myeloid DCs were defined by the surface expression profile CD11b+F4/80^{INT} and expressed as a proportion of total DCs; dots are individual mice; bars are means; data analysed by 1-way ANOVA with Sidak's multiple comparisons test; * $P \leq 0.05$. (F) Illustrative photomicrographs of Foxp3 IHC in indicated conditions at D9 post HDTV injection of the indicated constructs; scale bar 200µm. (G) Quantification of Foxp3-positive area per liver section, by IHC. Dots are individual mice; bars are means; data analysed by 1-way ANOVA with Sidak's multiple comparisons test; ** $P \leq 0.01$. (H) Putative model of COX2-dependent regulation of SASP composition and functionality. COX2 expression in RIS is C/EBPβ and NF-κB-dependent; amongst downstream products PGE$_2$ is able to bind EP4 receptors and modulate SASP expression or directly modulate immune cell functionality. Dotted lines represent indirect or uncertain pathways of regulation.
