# Supplementary material for: COX2 regulates senescence secretome composition and senescence surveillance through PGE2
Source: Cell Rep. 2021 Mar 16;34(11):108860. doi: 10.1016/j.celrep.2021.108860 (PMC7972992; doi:10.1016/j.celrep.2021.108860)
Supplement: Document S2. Article plus supplemental information [file mmc4.pdf]

Cell Reports

Article

COX2 regulates senescence secretome composition and senescence surveillance through PGE_2

Graphical Abstract

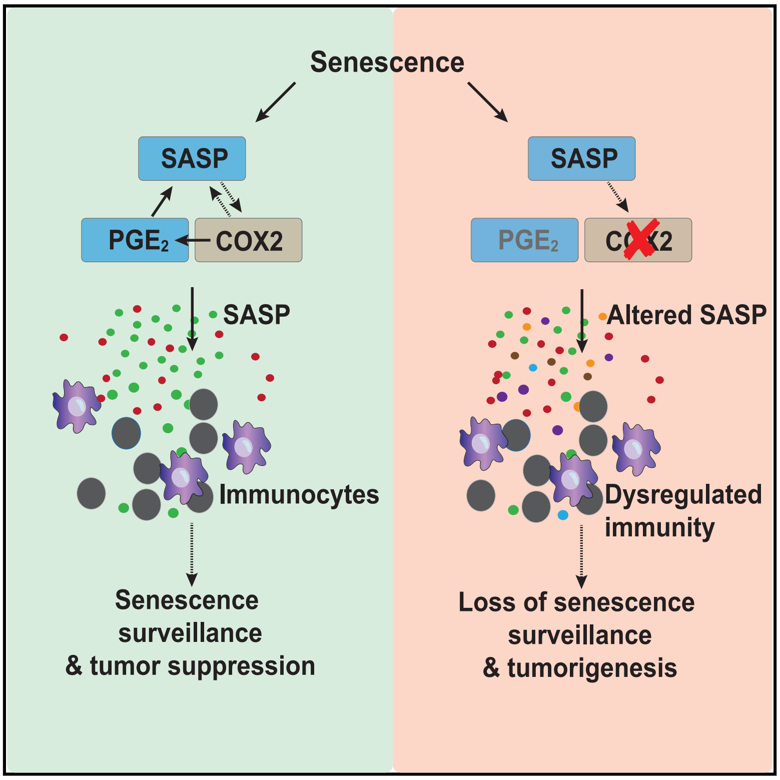

Authors

Susana Gonçalves, Kelvin Yin, Yoko Ito, ..., Andrew Young, Masashi Narita, Matthew Hoare

Correspondence

mwh20@cam.ac.uk

In brief

Gonçalves et al. identify COX2 as a regulator of senescence secretome composition through an autocrine feedback loop involving PGE_2 and EP4. During hepatocyte senescence, Cox2 is critical to tumor suppression, Cxcl1 expression, the immune microenvironment, and immune-mediated senescence surveillance, partially through PGE_2.

Highlights

- COX2 is upregulated in senescence and controls expression of multiple SASP components
- COX2 regulates SASP composition partly via autocrine feedback involving PGE_2 and EP4
- In hepatocyte senescence, Cox2 promotes senescence surveillance and tumor suppression
- Cox2 regulates the immune microenvironment of the liver, partly through PGE_2

Gonçalves et al., 2021, Cell Reports *34*, 108860
March 16, 2021 © 2021 The Author(s).
https://doi.org/10.1016/j.celrep.2021.108860

CellPress

Cell Reports

CellPress
OPEN ACCESS

Article

COX2 regulates senescence secretome composition and senescence surveillance through PGE_2

Susana Gonçalves,[1] Kelvin Yin,[1] Yoko Ito,[1] Adelyne Chan,[1] Ioana Olan,[1] Sarah Gough,[1] Liam Cassidy,[1] Eva Serrao,[1] Stephen Smith,[2] Andrew Young,[1] Masashi Narita,[1,3] and Matthew Hoare[1,4,5,*]

[1]CRUK Cambridge Institute, University of Cambridge, Cambridge CB2 0RE, UK

[2]Department of Pathology, University of Cambridge, Cambridge CB2 1QP, UK

[3]Tokyo Tech World Research Hub Initiative (WRHI), Institute of Innovative Research, Tokyo Institute of Technology, Yokohama, Kanagawa 226-0026, Japan

[4]Department of Medicine, University of Cambridge, Cambridge CB2 0QQ, UK

[5]Lead contact

*Correspondence: mwh20@cam.ac.uk

https://doi.org/10.1016/j.celrep.2021.108860

SUMMARY

Senescent cells trigger their own immune-mediated destruction, termed senescence surveillance. This is dependent on the inflammatory senescence-associated secretory phenotype (SASP), which includes COX2, an enzyme with complex roles in cancer. The role COX2 plays during senescence surveillance is unknown. Here, we show that during RAS-induced senescence (RIS), COX2 is a critical regulator of SASP composition and senescence surveillance *in vivo*. COX2 regulates the expression of multiple inflammatory SASP components through an autocrine feedback loop involving its downstream product, prostaglandin E2 (PGE_2), binding to EP4. During *in vivo* hepatocyte RIS, Cox2 is critical to tumor suppression, Cxcl1 expression, and immune-mediated senescence surveillance, partially through PGE_2. Loss of Cox2 in RIS dysregulates the intrahepatic immune microenvironment, with enrichment of immunosuppressive immature myeloid cells and $CD4^+$ regulatory T lymphocytes. Therefore, COX2 and PGE_2 play a critical role in senescence, shaping SASP composition, promoting senescence surveillance and tumor suppression in the earliest stages of tumorigenesis.

INTRODUCTION

In response to cellular stress, senescence acts as an intrinsic tumor suppressor mechanism, engaging a long-term cell-cycle arrest (Hoare and Narita, 2018). Although non-proliferative, senescent cells are metabolically active and have profound effects on the microenvironment through the senescence-associated secretory phenotype (SASP) (Hoare and Narita, 2018). The SASP, regulated by several chromatin-binding proteins, including RELA (Chien et al., 2011) and C/EBPβ (Kuilman et al., 2008), contains a range of cytokines, growth factors, and matrix-modifying enzymes previously shown to have differing and sometimes antagonistic functionality (Hoare and Narita, 2018). Therefore, it has become clear that rather than a singular secretome, there must exist sub-phenotypes to the SASP tailoring output to the cellular context (Hoare et al., 2016; Toso et al., 2014). The underlying basis for regulation of SASP composition is only starting to become clear (Hoare et al., 2016; Toso et al., 2014) but may afford therapeutic opportunities to promote senescent cell clearance.

Critical to the tumor suppressor function of senescence is the SASP-dependent immune-mediated elimination of senescent cells, which is termed senescence surveillance. Depending on the model studied, $CD4^+$ lymphocytes (Kang et al., 2011), $CD8^+$ lymphocytes (Ovadya et al., 2018), macrophages (Eggert et al., 2016; Kang et al., 2011; Lujambio et al., 2013) and natural killer (NK) cells (Sagiv et al., 2013) have been demonstrated to be crucial. NRAS-induced hepatocyte senescence leads to a $CD4^+$ T-lymphocyte and macrophage-dependent immune reaction (Kang et al., 2011). Immature myeloid cells (iMCs) are recruited in a Ccl2-dependent manner (Eggert et al., 2016) and develop into macrophages with effector functions to eliminate the senescent hepatocytes. This process can be subverted by cancer cells within the same microenvironment, preventing senescence surveillance (Eggert et al., 2016).

COX2 is an inducible cyclooxygenase that generates an array of downstream lipid mediators, including prostaglandins. COX2 has been implicated in the pathogenesis of several cancers (Bonavita et al., 2020; Hashemi Goradel et al., 2019), where it functions to drive apoptosis resistance, proliferation, angiogenesis, and inflammation. Among the downstream products of COX2, perhaps the best studied is prostaglandin E2 (PGE_2), with myriad roles in inflammation and immunity (Kalinski, 2012). Generally, most of the described functions of PGE_2 are immunosuppressive, leading to reduced cytolytic or phagocytic abilities in neutrophils, NK cells, and macrophages (Kalinski, 2012). In the developing adaptive immune response against established melanoma, PGE_2 is immunosuppressive; PGE_2 suppresses

This is an open access article under the CC BY license (http://creativecommons.org/licenses/by/4.0/).

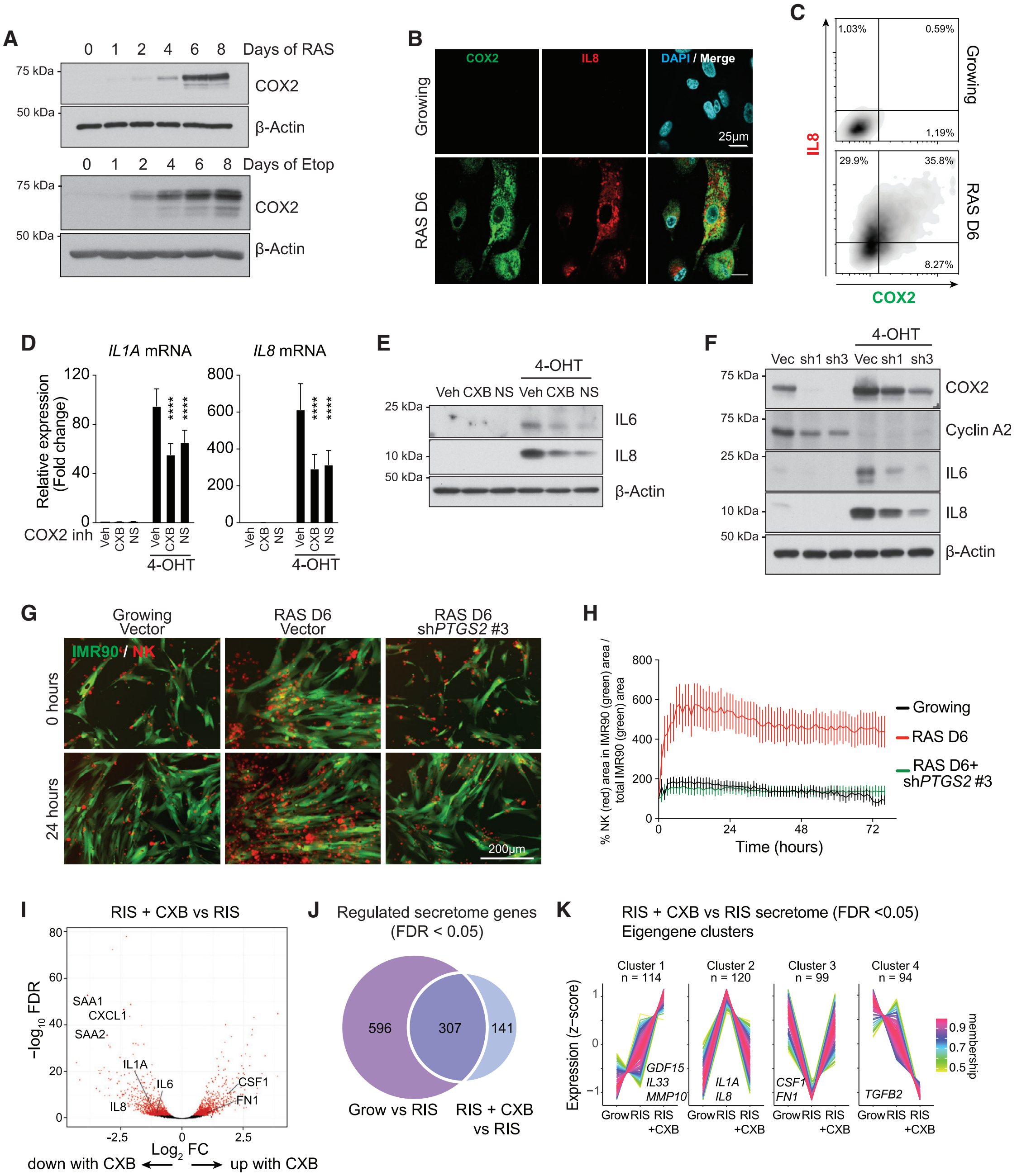

Figure 1. COX2 is upregulated in multiple forms of senescence and regulates SASP composition

(A) Time course of COX2 expression by immunoblotting during HRAS-induced senescence (RIS) (top) or DNA damage-induced senescence (DDIS) (bottom) induced by etoposide treatment.

(B and C) Representative immunofluorescence of COX2 and IL-8 (B) in growing or RIS ER:HRAS^{G12V} IMR90 cells (scale bars, 25 µm) with a contour density plot (C) of COX2/IL-8 expression from imaging cytometry of the same cells.

(D and E) Gene or protein expression in growing or RIS ER:HRAS^{G12V} IMR90 cells treated with vehicle, CXB, or NS398 was analyzed by qRT-PCR (D; n = 11 biologically independent experiments for all conditions; two-way ANOVA with Tukey's multiple comparisons test; values are mean ± SEM; ****$p \leq 0.0001$ versus RIS/vehicle condition) or immunoblotting for indicated proteins (E).

(F) Growing or RIS ER:HRAS^{G12V} IMR90 cells, expressing shRNAs against *PTGS2* or vector control, were analyzed for expression of indicated proteins by immunoblotting.

(G and H) Representative immunofluorescence (G) of co-cultures of CellTracker-Red-labeled NK (YT) cells with mVenus-labeled growing or RIS ER:HRAS^{G12V} IMR90s expressing the indicated sh*PTGS2* or vector control; scale bar, 200 µm.

(H) Quantification of NK (red signal) colocalization onto IMR90 cells (green signal) at the indicated time points. Line and whiskers represent mean ± SEM.

(legend continued on next page)

dendritic cell (DC)-dependent adaptive anti-tumoral immunity (Böttcher et al., 2018; Zelenay et al., 2015).

COX2 has been linked to cellular senescence previously. COX2 and downstream prostaglandins, such as PGE_2, are upregulated in replicative senescence in fibroblasts (Zdanov et al., 2007). COX2 is upregulated in oncogene-induced senescence *in vitro,* in a *Tlr2*-dependent manner (Hari et al., 2019), and in murine $Kras^{G12D}$-driven pancreatic intraepithelial neoplasia (PanIN), shown to contain senescent epithelial cells (Hingorani et al., 2003). Additionally, in both lung and oral fibroblasts, COX2-dependent PGE_2 has been shown to regulate the SASP component interleukin-6 (IL-6) (Dagouassat et al., 2013; Kabir et al., 2016).

Therefore, COX2, a suppressor of multiple immune mechanisms, particularly in established cancer, may be upregulated in senescence contemporaneously with the pro-immunogenic SASP. Whether COX2 plays a complementary or antagonistic role to the SASP and its role in immune-mediated senescence surveillance are largely unknown. We sought to investigate the *in vitro* and *in vivo* role of COX2 in senescence.

RESULTS

COX2 in senescence

To investigate the role of COX2 (encoded by the *PTGS2* gene) in senescence, we reanalysed mRNA-sequencing (mRNA-seq) data (Hoare et al., 2016) (GEO: GSE72407) from IMR90 human diploid fibroblasts (HDFs) undergoing RAS-induced senescence (RIS) or DNA damage-induced senescence (DDIS). *PTGS2* was significantly upregulated in both forms of senescence compared to growing cells (Figure S1A). Utilizing the same IMR90 cells expressing a 4-hydroxytamoxifen (4-OHT)-inducible form of oncogenic $HRAS^{G12V}$ (ER:$HRAS^{G12V}$), we found a significant upregulation of COX2 protein in both RIS and DDIS (Figure 1A); COX2 is also upregulated in two further HDF lines undergoing RIS (Figure S1B). We next studied Cox2 expression in murine $Kras^{G12D}$-driven PanIN, which was previously reported to show evidence of senescence (Collado et al., 2005; Hingorani et al., 2003). Cox2 is upregulated in the pre-malignant ductal epithelium compared to the normal pancreatic ductal epithelium (Figure S1C). Functionally, COX2 upregulation leads to an increase in the COX2 downstream product PGE_2 in both RIS and DDIS (Figure S1D). Therefore, consistent with previous studies (Dagouassat et al., 2013; Kabir et al., 2016; Zdanov et al., 2007), COX2 expression and activity were upregulated in multiple forms of senescence both *in vitro* and *in vivo*.

We reasoned that COX2 or its downstream products may form part of the pro-inflammatory SASP. Immunofluorescent staining showed colocalization of COX2 with interleukin-8 (IL-8), a chemokine and well-recognized part of the SASP (Kuilman et al., 2008; Figures 1B and 1C).

Inhibition of COX2 does not affect RIS induction

We next investigated the role of COX2 function during RIS using the specific COX2 inhibitors (COX2is) celecoxib (CXB) and NS398 (NS); these reduce PGE_2 back to baseline levels in RIS (Figure S1E). Unlike previous studies showing that COX2is modulated induction of replicative senescence (Dagouassat et al., 2013; Zdanov et al., 2007), COX2is did not affect RIS induction in IMR90. Compared to RIS cells, there were no differences in COX2i-treated RIS IMR90s in colony-forming ability (Figure S1F), senescence-associated β-galactosidase (SA β-gal) expression (Figures S1G and S1H), or bromodeoxyuridine (BrdU) incorporation (Figure S1I). Therefore, in the context of RIS, COX2 function does not regulate senescence arrest.

Inhibition of COX2 blunts SASP expression and functionality

Unlike other markers of senescence, COX2is led to changes in several SASP genes. Treatment with COX2is during RIS led to downregulation of multiple SASP components, including *IL1A*, IL-6, and IL-8, at both the mRNA (Figure 1D) and protein levels (Figure 1E). To confirm that this was not an off-target effect of COX2is, we used small hairpin RNA (shRNA)-mediated knockdown in RIS, which led to an effective, but incomplete, reduction in *PTGS2* expression (Figure 1F) and activity (Figure S1J). Similarly to COX2is, knockdown of COX2 was associated with downregulation of IL-6 and IL-8 but no change in cyclin A2 expression (Figures 1F and S1K). Therefore, inhibition of COX2 expression or activity during RIS leads to downregulation of several pro-inflammatory SASP genes.

This association of *PTGS2* with inflammatory cytokine expression extends to established cancer; analysis of mRNA-seq data from the Cancer Genome Atlas (TCGA) dataset showed positive correlations of *PTGS2* expression with *IL1B*, *IL6*, *IL8*, and *CXCL1* in all cancer types (Figure S1L).

To demonstrate that COX2 inhibition or subsequent loss of SASP components is associated with loss of SASP-regulated functionality, we utilized an *in vitro* chemotaxis assay; here, the RIS SASP attracts NK cells to the senescent cell (Tasdemir et al., 2016). Co-culture of RIS IMR90s with the YT NK cell line drives labeled NK cells to colocalize with RIS, but not growing cells, in a SASP-dependent manner (Tasdemir et al., 2016). Consistently, RIS HDFs attracted YT cells within hours of co-culture, whereas knockdown of COX2 in the RIS HDFs completely abrogated this chemotaxis (Figures 1G and 1H).

Therefore, COX2 regulates the expression of several SASP components and controls downstream immune cell

(I) Growing or RIS ER:$HRAS^{G12V}$ IMR90 cells treated with vehicle or CXB were harvested for mRNA-seq (n = 8 biologically independent replicates for all conditions). Volcano plot shows $\log_2$ fold change in gene expression against the $-\log_{10}$FDR in RIS/CXB compared to RIS alone; red dots are genes with significant differential expression (DE; FDR < 0.05).

(J) Venn diagram of numbers of DE secretome genes demonstrating 903 secretome genes DE in RIS compared to growing cells; of these, 307 genes are also DE in the RIS/CXB condition compared to RIS/vehicle.

(K) Eigengene clusters of *Z*-score-normalized gene expression were plotted for secretory genes with similar expression patterns across conditions (n = 1,132), demonstrating four clusters of gene behavior; exemplar genes from each cluster are listed.

See also Figure S1.

functionality, either directly or through loss of these SASP components.

COX2 regulates SASP composition

To understand whether COX2 regulates a small number of SASP components or the SASP more generally, we performed mRNA-seq of RIS IMR90s, with or without CXB-mediated COX2is. Among genes significantly downregulated by CXB in the context of RIS, there were a number of SASP components, including *IL6*, *IL8*, *CXCL1*, and *SAA1*. SAA1 was recently shown to be a TLR2-dependent component of the SASP (Hari et al., 2019; Figure 1I).

On filtering the dataset for secretome genes (Hoare et al., 2016), we found that RIS leads to differential expression (DE; false discovery rate [FDR] < 0.05) of 903 secretome genes compared to growing cells. Of these, 307 (34%) are also significantly differentially expressed in RIS with CXB compared to RIS alone (Figure 1J). This suggests that COX2 functions to regulate large parts of the SASP. Consistently, we found that canonical members of the SASP, such as *IL1A*, *IL8*, and *CXCL1*, were COX2 dependent but that expression of many other SASP components, such as *MMP1*, *MMP3*, and *PLAUR*, were COX2 independent (Figure S1M). Indeed, some components, such as *MMP10*, show augmented expression with CXB (Figure S1N).

To better understand the nature of the 448 COX2-dependent SASP genes, we identified eigengene clusters with similar behavior between conditions (Figure 1K; Table S1). Cluster 2 contains multiple genes from the classical pro-inflammatory SASP, such as *IL1A*, *IL6*, *IL8*, and *SAA1*(Hari et al., 2019), with upregulation in RIS but downregulation in RIS with CXB (Figure 1K). Transcription factor motif analysis demonstrated a predicted dependence of cluster 2 genes upon C/EBPβ and nuclear factor κB (NF-κB) pathways (Figure S1O). Reciprocally, cluster 3 genes, such as *FN1* and *CSF1* (encoding fibronectin and macrophage colony-stimulating factor [M-CSF], respectively), showed repression in RIS but near-complete rescue with CXB (Figure 1K). Therefore, there are complex but broad patterns of COX2-dependent SASP gene regulation.

COX2 and the SASP are regulated in similar fashion

The upstream regulation of the SASP is complex, but previous studies have demonstrated that the canonical NF-κB component RELA (Chien et al., 2011) and C/EBPβ (Kuilman et al., 2008) cooperatively regulate the SASP. COX2 is regulated by C/EBPβ, as shRNA-mediated knockdown of *CEBPB* in RIS leads to loss of not only SASP components, such as IL-8, but also COX2 expression and activity (Figures S2A and S2B). Further, overexpression of the transcriptionally active form of C/EBPβ, LAP*, leads to an upregulation of COX2 (Figure S2C). Similarly, inhibition of NF-κB function during RIS, through co-expression of an IκBα$^{S32A / S36A}$ "super-repressor" (SR), abrogates IL-6, IL-8, and COX2 expression (Figure 2A), reinforcing previous studies demonstrating COX2 expression to be NF-κB dependent in other contexts (Gallois et al., 1998). Consistent with our recent findings that NOTCH1 dominantly regulates SASP composition (Hoare et al., 2016), ectopic expression of the transcriptionally active NOTCH1 intracellular domain (N1ICD) in RIS repressed *IL1A*, *PTGS2*, and PGE_2 expression (Figures S2D and S2E). Therefore, COX2 is similarly regulated to other members of the classical SASP in RIS.

COX2 regulates SASP composition through PGE_2 and EP4

COX2 activity produces an array of downstream mediators. To understand how COX2 regulates SASP composition, we hypothesized that it could generate an autocrine feedback loop, as previously demonstrated for IL-8 (Acosta et al., 2008; Kuilman et al., 2008). Among COX2 products, PGE_2 is generated at high concentrations and has been linked with replicative senescence previously (Dagouassat et al., 2013). Therefore, we investigated whether PGE_2 could contribute to COX2-dependent SASP modulation.

We utilized CXB during RIS to inhibit SASP gene expression before treating the cells with exogenous PGE_2, downstream of COX2is. Whereas CXB treatment inhibits *IL1B* and *IL8* expression but augments *CSF1* expression, treatment with PGE_2 rescues all of these changes at both the mRNA (Figure 2B) and protein (Figure S2F) levels. Inhibition of NF-κB during RIS leads to loss of both COX2 and SASP gene expression (Figure 2A); exogenous PGE_2 partially rescues SASP-component expression (Figures 2C and 2D) in this context. However, exogenous PGE_2 did not rescue *IL1A* and *IL8* expression in the context of N1ICD-mediated SASP repression (Figure S2G).

PGE_2 binds to four E-type prostaglandin (EP) receptors encoded by *PTGER* genes. Our previous mRNA-seq data from RIS and DDIS IMR90s showed that only EP2 (*PTGER2*) and EP4 (*PTGER4*) are expressed in growing, RIS, or DDIS IMR90s (Figure S2H). To study which receptor is crucial for PGE_2-mediated SASP regulation, we utilized specific pharmacological inhibitors of different EP receptors (Figure 2E). Inhibitors of EP4, but not EP2, are able to phenocopy loss of COX2, with reduction of IL-8 expression (Figures 2F and S2I). Consistently, whereas exogenous PGE_2 is able to rescue IL-8 expression in CXB-treated RIS cells, PGE_2 is unable to rescue IL-8 expression in EP4-inhibitor-treated RIS cells, suggesting that PGE_2, through EP4, regulates IL-8 in an autocrine feedback loop (Figure 2G).

Cox2 is critical for senescence surveillance and tumor suppression *in vivo*

It has become clear that immune-mediated senescence surveillance is critical to the tumor suppressive function of senescence *in vivo*. We hypothesized that Cox2 and Cox2-dependent SASP regulation would be critical in senescence surveillance.

Hydrodynamic tail vein (HDTV)-injected delivery of transposable elements containing oncogenic NRAS^{G12V} leads to hepatocyte RIS in 6 days, with a subsequent CD4^{+} T-lymphocyte- and macrophage-dependent immune reaction (Eggert et al., 2016; Kang et al., 2011), driving clearance of RIS hepatocytes from days (D) 6 to 12 post-HDTV injection (Hoare et al., 2016). We modified the construct containing the NRAS^{G12V} transposon to express Cre-recombinase outside of the transposon (Figure S3A). Therefore, after HDTV injection this leads to stable integration and expression of NRAS but transient episomal expression of Cre. We injected this into *Ptgs2*$^{fl/fl}$ mice, which led to RIS hepatocytes with Cre-dependent knockout of *Ptgs2*. The control constructs contained nonfunctional NRAS$^{G12V / D38A}$ (Kang et al., 2011) with Cre and oncogenic NRAS^{G12V} without Cre (Figure 3A). Injection of Cre-expressing constructs led to expression of Cre (Figure S3B) and an abrogation of

CellPress
OPEN ACCESS

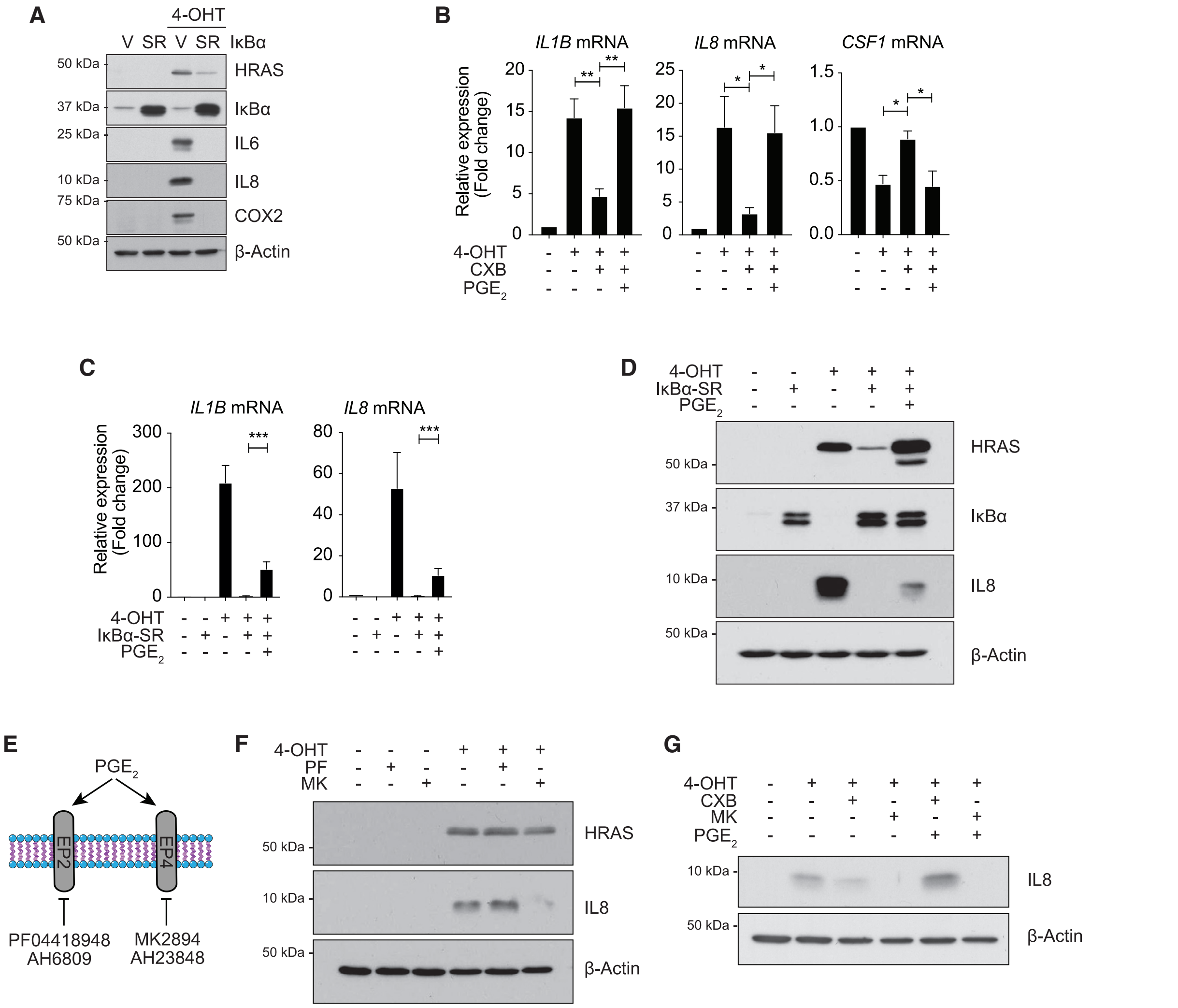

Figure 2. Upstream regulation of COX2 and downstream regulation of the SASP through PGE_2

(A) RIS-induced COX2 expression is regulated by NF-κB. Growing or RIS ER:HRAS^{G12V} IMR90 cells, expressing an IκBα super-repressor (SR; IκBα$^{S32A\,/\,S36A}$) or vector control, were analyzed for expression of indicated proteins by immunoblotting.

(B) Expression of *IL1B*, *IL8*, and *CSF1* in growing or RIS ER:HRAS^{G12V} IMR90 cells treated with vehicle or CXB and vehicle or PGE_2 (10 μM) were analyzed by qRT-PCR; n $\geq$ 4 biologically independent replicates for all conditions; values are mean ± SEM; statistical analysis by one-way ANOVA with Sidak's multiple comparisons test; *p $\leq$ 0.05, **p $\leq$ 0.01.

(C and D) Growing or RIS ER:HRAS^{G12V} IMR90 cells, expressing the IκBα-SR or vector control and treated with vehicle or PGE_2 were analyzed by qRT-PCR (C) or immunoblotting (D); n = 6 biologically independent replicates for all conditions; values are mean ± SEM; statistical analysis by 1-way ANOVA with Sidak's multiple comparisons test; ***p $\leq$ 0.001.

(E) PGE_2 binds to EP receptors; in subsequent experiments, we utilized the EP2 inhibitors PF04418948 (PF; 1 μM) and AH6809 (10 μM) or the EP4 inhibitors MK2894 (MK; 1 μM) and AH23848 (10 μM).

(F) Growing or RIS ER:HRAS^{G12V} IMR90 cells, treated with vehicle, PF, or MK, were analyzed for expression of indicated proteins by immunoblotting.

(G) Growing or RIS ER:HRAS^{G12V} IMR90 cells, treated with vehicle, CXB, or MK and vehicle or PGE_2, were analyzed for expression of indicated proteins by immunoblotting, demonstrating that while PGE_2 can rescue IL-8 expression when COX2 is inhibited, this is prevented when EP4 is inhibited.

See also Figure S2.

RIS-dependent upregulation of PGE_2 expression (Figure S3D), consistent with Cre-mediated recombination and knockout of Cox2, only in the NRAS-transduced hepatocytes.

We studied the temporal dynamics of RIS hepatocyte clearance in this model. All conditions had similar levels of NRAS-expressing hepatocytes at day 6 post-HDTV injection. Consistent with previous data (Hoare et al., 2016; Kang et al., 2011), hepatocytes transduced with oncogenic NRAS^{G12V} alone are progressively cleared from the liver. However, *Ptgs2* knockout in the context of RIS completely prevents this time-dependent clearance of RIS hepatocytes (Figures 3B, 3C, and S3C). Importantly, and consistent with our *in vitro* data, this is not due to senescence bypass, as RIS and RIS/Cox2-knockout hepatocytes showed similar low levels of 5-ethynyl-2′-deoxyuridine (EdU) incorporation (Figure S3E).

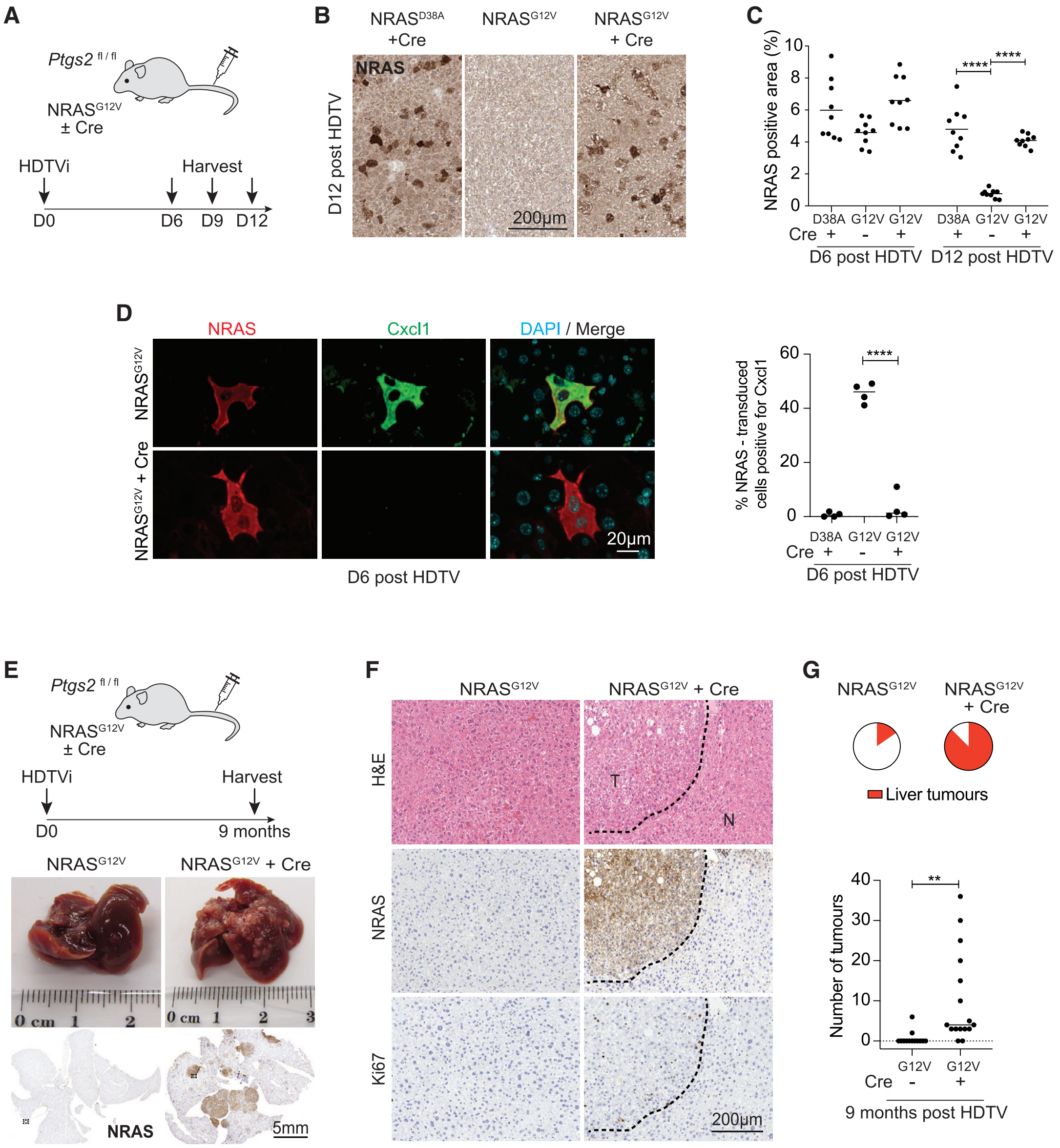

Figure 3. Cox2 regulates senescence surveillance and tumor suppression *in vivo*

(A) Experimental outline. *Ptgs2*$^{fl/fl}$ mice underwent hydrodynamic tail vein (HDTV) injection of transposons containing inactive NRAS$^{G12V / D38A}$ or oncogenic NRAS^{G12V} to induce hepatocyte RIS. In some conditions, the injected plasmids also contained Cre recombinase, leading to *Ptgs2* knockout in the same cells. Mice were harvested at day 6, 9, or 12 post-HDTV injection.

(B and C) Temporal dynamics of NRAS^{+} cell clearance after RIS induction with or without *Ptgs2* knockout. (B) Illustrative photomicrographs of NRAS immunohistochemistry (IHC) in indicated conditions. (C) Quantification of NRAS-positive area by IHC at day 12 after HDTV injection. Dots represent individual mice, and bars indicate means. Day 12 data were analyzed by one-way ANOVA with Sidak's multiple comparisons test; ****p $\leq$ 0.0001.

(D) Representative immunofluorescence images (left) of NRAS and Cxcl1 in mouse livers at day 6 post-HDTV injection of the indicated constructs; scale bar 20 μm and quantification of the number of NRAS+ hepatocytes that were also positive for Cxcl1 staining (right) in the indicated conditions. Dots represent individual mice, and bars indicate means. Data were analyzed by one-way ANOVA with Sidak's multiple comparisons test; ****p $\leq$ 0.0001.

(E–F) Loss of *Ptgs2* in RIS abrogates tumor suppression. (E) Top: experimental outline; *Ptgs2*$^{fl/fl}$ mice underwent HDTV injection of NRAS^{G12V}-transposons with or without Cre recombinase and were harvested at 9 months post-injection. Middle: example images of gross liver pathology of one mouse from each cohort revealing multiple liver lesions in the NRAS^{G12V}-Cre injected livers. Bottom: illustrative photomicrographs of NRAS IHC in the same livers. Scale bar, 5 mm. Dotted lines are the regions chosen for high-magnification images in (F).

(F) Illustrative photomicrographs of H&E staining (top), NRAS (middle), and Ki67 (bottom) IHC in indicated conditions. T, tumor; N, normal liver. Scale bar, 200 μm.

(G) Pie charts (top) demonstrating proportion of each cohort with liver tumors at autopsy (n $\geq$ 13 individual mice per condition) and dot-plot showing numbers of liver tumors per liver. Dots represent individual mice, and bars indicate means. Data were analyzed by unpaired t test; **p $\leq$ 0.01.

See also Figure S3.

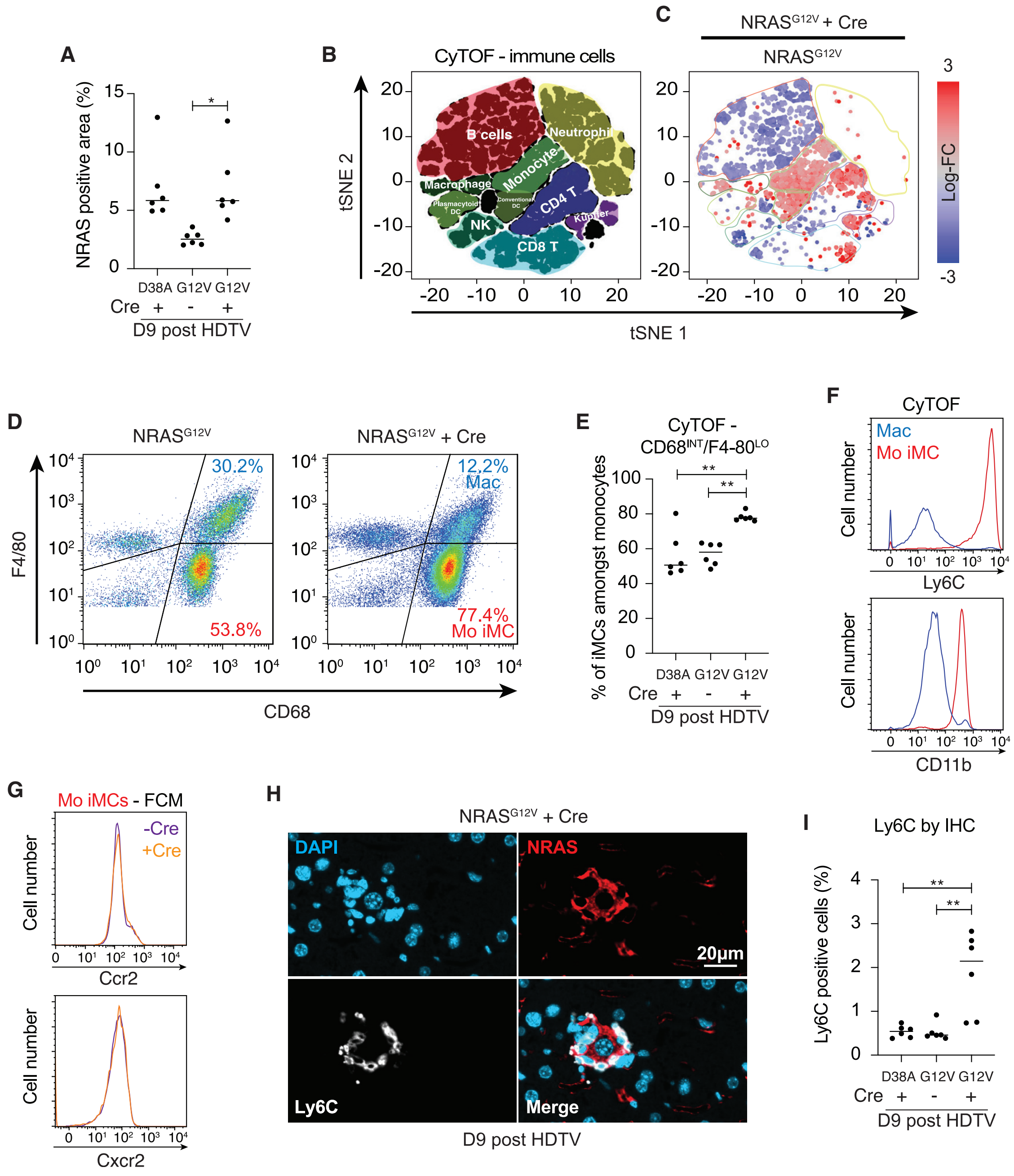

Figure 4. Cox2 controls the intrahepatic immune microenvironment during senescence surveillance

(A) *Ptgs2*$^{fl/fl}$ mice underwent HDTV injection of transposons containing inactive NRAS$^{G12V / D38A}$ or oncogenic NRAS^{G12V} to induce hepatocyte RIS with or without Cre recombinase before harvest at day 9 post-injection. Quantification of NRAS-positive area by IHC is shown. Dots indicate individual mice, and bars represent means. Data were analyzed by one-way ANOVA with Sidak's multiple comparisons test; $^{*}p \leq 0.05$.

(B) **t**-distributed stochastic neighbor embedding (t-SNE) plots of multiplexed intrahepatic immune cell mass cytometry data from the same 18 mice in (A) (n = 6 per condition) using 23 metal-tagged antibodies against immunocyte surface markers (see Table S2).

(B and C) Hyperspheres of immune cell phenotypes were identified by typical surface marker expression (B) and colored according to log-fold change in abundance (C) between NRAS^{G12V} versus NRAS^{G12V} + Cre conditions.

(D and E) Live lineage-negative, non-granulocytic/non-DC immune cells were gated as in Figure S4C, before analysis of different monocyte populations based on CD68 and F4/80 expression, identifying monocytic immature myeloid cells (Mo iMCs) as CD68^{INT} and F4/80^{LO}; example dot-plots of monocyte populations from NRAS^{G12V} and NRAS^{G12V} + Cre-injected mice are shown in (D) with quantification in (E). Dots indicate individual mice, and bars represent means. Data were analyzed by one-way ANOVA with Sidak's multiple comparisons test; $^{**}p \leq 0.01$.

(F) Histograms from CyTOF data demonstrating expression of Ly6C and CD11b on iMCs and macrophages (including Kupffer cells, CD68^{HI}/F4/80^{HI}) from NRAS^{G12V} + Cre-injected mouse liver.

(legend continued on next page)

Our *in vitro* data suggest that loss of Cox2 in RIS leads to reduced SASP expression. Co-staining of transduced livers for NRAS and Cxcl1, a component of the hepatocyte SASP (Eggert et al., 2016), shows that RIS hepatocytes upregulate Cxcl1; in similar animals with RIS/Cox2 knockout, this upregulation is not observed (Figure 3D). Therefore, consistent with our *in vitro* data, Cox2 is critical for the expression of the SASP component Cxcl1 and immune-mediated senescence surveillance *in vivo*.

Previous data in the same model showed that loss of immune-mediated senescence surveillance leads to long-term tumorigenesis (Kang et al., 2011). Therefore, we injected *Ptgs2*$^{fl/fl}$ animals with oncogenic NRAS^{G12V}, with or without Cre, and followed them for 9 months. At this point, the majority of NRAS-injected Cox2-knockout animals develop macroscopic liver tumors (Figures 3E–3G) with microscopic features of hepatocellular carcinoma (HCC) (Figure 3F), whereas most NRAS-injected animals with normal Cox2 function have no tumors (Figure 3G). Therefore, Cox2 controls Cxcl1 expression, senescence surveillance, and long-term tumor suppression in the context of RIS *in vivo*.

Cox2 regulates the intrahepatic immune microenvironment in senescence surveillance

We hypothesized that the loss of senescence surveillance could be due to alterations in the Cox2-dependent SASP, leading to changes in the intrahepatic immune microenvironment. We studied mice at day 9 post-HDTV injection, a time point previously shown to be critical to this immune surveillance (Eggert et al., 2016; Hoare et al., 2016; Kang et al., 2011). At this time point, the loss of senescence surveillance is already evident with increased RIS hepatocytes in NRAS/Cox2 knockout compared to NRAS alone (Figures 4A and S4A).

We multiplexed intrahepatic immune cells from 18 mice and performed deep immunophenotyping using mass cytometry with 23 metal-tagged antibodies against immune markers (Figure 4A; Table S2); this allows identification and quantification of relative immune subset enrichment in the three conditions. Utilizing dimensionality reduction, NRAS/Cox2 knockout is associated with a significant enrichment of monocyte populations (Figures 4B and 4C) expressing CD68, F4/80, Ly6C, and CD11c (Figure S4B), as well as a subset of the CD3^{+}CD4^{+} T-lymphocyte population (Figures 4B and 4C).

To confirm the identity of the Cox2-regulated monocyte population, we gated on non-granulocytic/non-dendritic myeloid cells (Figure S4C). Within this population, Cox2 knockout is associated with a significant enrichment of myeloid cells with an immature (CD68^{INT}, F4/80^{LO}) rather than mature macrophage phenotype (CD68^{HI}, F4/80^{HI}) (Figures 4D and 4E). This population also displayed high levels of Ly6C and CD11b expression (Figure 4F), consistent with an iMC population with immunosuppressive properties, as defined by Eggert et al. in the same model (Eggert et al., 2016). Although the iMCs were more abundant when Cox2 was lost, they had a similar surface phenotype between conditions; intrahepatic iMCs were positive for Ccr2 (Eggert et al., 2016) and Cxcr2 in both NRAS and NRAS/Cox2-knockout conditions (Figure 4G).

We confirmed this enrichment of Ly6C^{+} cells (Figures 4H and 4I), but not CD68^{+} cells (Figure S4D), using immunohistochemistry (IHC) as an orthogonal analysis of absolute cell number per liver area rather than as a proportion of intrahepatic immune cells. Spatially, it was apparent that in RIS/Cox2-knockout livers, these Ly6C^{+} cells formed tight clusters around the RIS hepatocytes (Figure 4H).

COX2 has previously been demonstrated to modulate DC recruitment and behavior (Böttcher et al., 2018; Zelenay et al., 2015) in the microenvironment. We found enrichment and spatial localization of CD11c^{+} DCs in the immune cell clusters around the RIS hepatocytes in the context of Cox2 knockout (Figures S4E and S4F). Analysis of DC subtype from our cytometric time-of-flight mass spectrometry (CyTOF) data showed that knockout of Cox2 is associated specifically with enrichment of CD11b^{+} F4/80^{INT} myeloid DCs (Figures S4G and S4H).

Investigation of the surface phenotype of the CD4^{+} lymphocytes enriched in the context of NRAS/Cox2 knockout showed that they had a CD25^{HI}/CD127^{LO} phenotype consistent with immunosuppressive CD4^{+} regulatory T cells (Treg cells) (Figure S4I). This enrichment was confirmed by a significant increase in FoxP3^{+} cells seen at IHC (Figures S4J and S4K). Therefore, Cox2 in RIS hepatocytes contributes to the repression of recruitment or maturation of two suppressive immune cell populations, iMCs and CD4^{+} Treg cells, and a skewing of the intrahepatic DC population.

Cox2 partially regulates senescence surveillance through PGE_2

Our *in vitro* data showed that COX2 could regulate SASP composition through PGE_2. We hypothesized that exogenous PGE_2 could rescue senescence surveillance in RIS when Cox2 was knocked out. Therefore, we injected mice undergoing hepatocyte RIS with dimethyl-PGE_2 (dmPGE_2), a synthetic derivative of PGE_2 with a longer half-life (Figure 5A). As before, Cox2 knockout in RIS leads to impaired senescence surveillance at day 9 post-HDTV injection (Figures 5B and 5C). Exogenous dmPGE_2 leads to a partial rescue, with significantly fewer RIS hepatocytes remaining at day 9 (Figures 5B and 5C). Consistent with the *in vitro* data, loss of Cox2 leads to loss of Cxcl1 expression in RIS hepatocytes, but exogenous dmPGE_2 treatment partially rescues Cxcl1 expression (Figure 5D).

We utilized a modified 16-marker mass cytometry panel, particularly targeting myeloid cells, to probe the intrahepatic immune microenvironment in these mice. Analysis of 15 mice across the three conditions again showed that RIS/Cox2 knockout was associated with increased abundance of monocytes, including CD68^{INT} F4/80^{LO} Ly6C^{+} iMCs (see population 2, Figures S5A and S5B), expressing high levels of Cx3cr1

(G) Histograms from flow cytometry demonstrating expression of Ccr2 and Cxcr2 on iMCs from NRAS^{G12V} and NRAS^{G12V} + Cre-injected mouse livers.
(H and I) Expression of Ly6C in the liver by IHC in same mice as in (A); example fluorescence IHC from NRAS^{G12V} + Cre-injected mouse demonstrating Ly6C^{+} cells surrounding an NRAS-positive hepatocyte in (H). Scale bar, 20 μm. (I) Quantification of Ly6C^{+} cells. Dots indicate individual mice, and bars represent means. Data were analyzed by one-way ANOVA with Sidak's multiple comparisons test; $^{**}p \leq 0.01$.
See also Figure S4.

CellPress
OPEN ACCESS

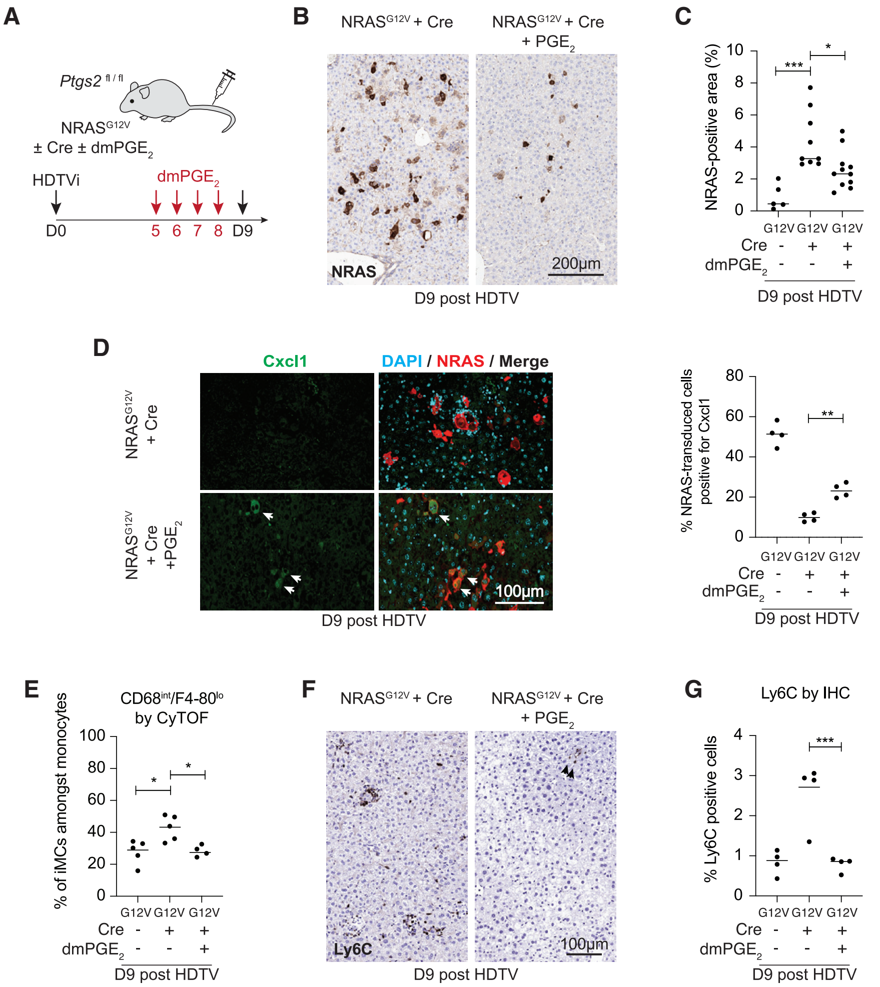

Figure 5. PGE_2 partially rescues Cox2-dependent immune-mediated senescence surveillance

(A) Experimental outline; *Ptgs2*$^{fl/fl}$ mice underwent HDTV injection of transposons containing oncogenic NRAS^{G12V} with or without Cre recombinase, leading to *Ptgs2* knockout in the same cells. Mice received daily intra-peritoneal injections of either vehicle or dimethyl-PGE_2 (dmPGE_2; 200 µg/kg) between day 5 and day 8 post-injection. Mice were harvested at day 9 after the HDTV injection.

(B) Illustrative photomicrographs of NRAS IHC in indicated conditions.

(C) Quantification of NRAS-positive area by IHC. Dots indicate individual mice, and bars represent means. Data were analyzed by one-way ANOVA with Sidak's multiple comparisons test; *$p \leq 0.05$, ***$p \leq 0.001$.

(D) Representative immunofluorescence images of NRAS and Cxcl1 in mouse livers at day 9 post-HDTV injection of the indicated constructs. Scale bar, 100 µm. White arrowheads demonstrate same Cxcl1$^+$ cells between images. Quantification of the number of NRAS$^+$ hepatocytes that were also positive for Cxcl1 staining in the indicated conditions is shown. Dots indicate individual mice, and bars represent means. Data were analyzed by one-way ANOVA with Sidak's multiple comparisons test; **$p \leq 0.01$.

(E) Mass cytometry data showing live, lineage-negative, non-granulocytic/non-DC immune cells gated as in Figure S6C, before analysis of different monocyte populations based on CD68 and F4/80 expression, identifying iMCs as CD68^{INT} and F4/80^{LO}. Dots indicate individual mice, and bars represent means. Data were analyzed by one-way ANOVA with Sidak's multiple comparisons test; *$p \leq 0.05$.

(F and G) Expression of Ly6C in the liver by IHC in same mice used for mass cytometry; (F) example photomicrographs of Ly6C IHC from indicated mice demonstrating clusters of Ly6C$^+$ cells surrounding NRAS-positive hepatocytes in NRAS^{G12V} + Cre are mostly abrogated in mice treated with dmPGE_2. Arrowheads show residual Ly6C$^+$ cells. Scale bar, 100 µm. (G) quantification of Ly6C$^+$ cells. Dots indicate individual mice, and bars represent means. Data were analyzed by one-way ANOVA with Sidak's multiple comparisons test; ***$p \leq 0.001$.

See also Figure S5.

(Figure S5C). This accumulation is reversed when mice were treated with dmPGE_2 (Figure 5E). Consistently, dmPGE_2 completely reverses the accumulation of Ly6C$^+$ cells within the liver, and immune clusters around RIS hepatocytes are not seen on IHC (Figures 5F and 5G). Although numerically this Ly6C$^+$ iMC population was dependent upon PGE_2, there are no clear phenotypic differences in this population between conditions, with no change in surface expression of Cx3cr1, Csf1r (CD115), Siglec1 (CD169), Mrc1 (CD206), or VSig4 on Ly6C$^+$ iMCs between RAS/Cox2 knockouts and similar mice injected with dmPGE_2.

In addition to regulating the abundance of iMCs within the liver, administration of dmPGE_2 completely reversed the accumulation of DCs (Figure S5D), the specific accumulation of myeloid DCs (Figure S5E), and FoxP3$^+$ regulatory T cells (Figures S5F and S5G) associated with Cox2-knockout in RIS. Therefore, Cox2, partially through PGE_2, is able to regulate Cxcl1 expression, the immune microenvironment, and senescence surveillance within the liver.

DISCUSSION

Altogether, our data suggest that COX2 plays a significant role in the regulation of both SASP composition and its downstream functionality. Therefore, COX2 can be added to the complex web of factors demonstrated to regulate the SASP (Hoare and Narita, 2018). Although COX2 is able to modulate SASP composition, the pathways downstream of PGE_2 and EP4 remain unknown. As COX2 modulates *IL1A* transcription, which was previously implicated as an upstream SASP regulator (Orjalo et al., 2009), presumably, COX2 signaling must converge on transcriptional regulators such as GATA4 (Kang et al., 2015), C/EBPβ (Acosta et al., 2008; Kuilman et al., 2008), or NF-κB (Chien et al., 2011; Figure S5H). Previous data from

macrophages show that PGE_2 is able to drive C/EBPβ expression and activity through EP4 and PKA (Na et al., 2015), suggesting a plausible mechanism.

Importantly, in addition to regulation of SASP composition, we have demonstrated that Cox2 is important in the non-autonomous functionality of senescent cells and critical for immune-mediated senescence surveillance and tumor suppression *in vivo*. Loss of Cox2 in RIS is associated with an accumulation of immunosuppressive immune cells within the liver, including iMCs, previously implicated in evasion of senescence surveillance (Eggert et al., 2016). An open question that remains to be answered is whether the tumorigenesis that we observed is be mediated through (1) autonomous senescence bypass, (2) non-autonomous stimulation of tumorigenesis by senescent cells with a modified SASP, (3) bypass of immune-mediated senescence surveillance, or (4) a pro-tumorigenic effect of the immune cells that were recruited to the liver when Cox2 was lost. Although our data suggest that option 3 is most likely, further research will be required to define this. However, in the context of hepatocyte RIS, Cox2 and PGE_2 are immunostimulatory and anti-tumorigenic, either directly or indirectly.

Much of the previous literature has focused on the immunosuppressive effects of COX2 and PGE_2 signaling (Bonavita et al., 2020; Böttcher et al., 2018; Kalinski, 2012; Loo et al., 2017; Zelenay et al., 2015). In the context of HCC, senescent hepatic stellate cells (HSCs) produce COX2 and PGE_2; these were immunosuppressive, through inhibition of immune cell production of type 1 cytokines and repression of intrahepatic $CD103^+$ DC activity (Loo et al., 2017). Similarly, Cox2-dependent PGE_2 can modulate the secretome of *Braf*^{V600E}-expressing melanoma cells; loss of *Cox2* was associated with reduced *Il6* and *Cxcl1*, among other factors, consistent with our findings (Zelenay et al., 2015). However, in their model, PGE_2 was directly immunosuppressive through repression of DC (Böttcher et al., 2018; Zelenay et al., 2015) or NK cell recruitment (Böttcher et al., 2018) to the tumors, thereby contributing to immune escape. It is unclear how to reconcile these opposing functions of Cox2-dependent PGE_2 in promotion (in our study) or repression (in other studies) of anti-tumoral immunity. Whether this reflects differing roles of Cox2-dependent PGE_2 in tumor initiation and progression or the differing roles of indirect regulation of cytokine production and direct non-autonomous functionality will require further investigation.

While COX2-dependent PGE_2 was important in SASP regulation, our data suggest that other downstream products of Cox2 are likely to be important. Exogenous PGE_2 was able to completely rescue the changes in iMCs, DC, and Treg cell numbers associated with Cox2 knockout but only partially rescue the immune-mediated surveillance of RIS hepatocytes. This suggests that either non-enzymatic activity of COX2 or more likely other downstream products, of which PGE_2 is but one of many, are also crucial. In the previously mentioned study of HCC, Cox2 expression in HSCs drives an increase in several downstream Cox2 products within the liver, including PGD_2, PGE_2, and PGF_{2A} (Loo et al., 2017). However, only PGE_2 modulated immune cell functionality *in vitro*, and PGE_2-specific EP4 antagonists were sufficient to promote anti-tumoral immunity *in vivo*. Clearly, other studies clarifying the breadth and functional effects of senescence-associated lipid mediators in different contexts will be crucial.

STAR★METHODS

Detailed methods are provided in the online version of this paper and include the following:

- KEY RESOURCES TABLE
- RESOURCE AVAILABILITY
  - Lead contact
  - Materials availability
  - Data and code availability
- EXPERIMENTAL MODELS
  - *In vitro* cell culture
  - Mouse lines and husbandry
- METHOD DETAILS
  - Compounds
  - Vectors
  - *In vivo* vectors and cloning of Transposon-vectors with Cre-recombinase
  - NK-cell chemotaxis experiments
  - Expression profiling by mRNA sequencing and analysis
  - Sequencing alignment
  - Differential expression analysis
  - Gene trajectories
  - Venn Diagram
  - Enrichment
  - BrdU incorporation, colony formation and SA-β-galactosidase assays
  - Laser scanning cytometry
  - mRNA expression by quantitative RT-PCR
  - Protein expression by immunoblotting and immunofluorescence
  - Prostaglandin E2 ELISA
  - Mouse maintenance and experiments
  - Immunohistochemistry
  - Mass-cytometry and data analysis
  - Flow-cytometry and data analysis
  - TCGA analysis
- QUANTIFICATION AND STATISTICAL ANALYSIS

SUPPLEMENTAL INFORMATION

Supplemental Information can be found online at https://doi.org/10.1016/j.celrep.2021.108860.

ACKNOWLEDGMENTS

We thank all members of the Narita laboratory for helpful discussions and staff of the Cancer Research UK Cambridge Institute core facilities for technical support. We are grateful to Garret FitzGerald for the *Ptgs2*$^{fl/fl}$ mice and Jesus Gil for the ESF cell line. This work was supported by a CRUK Advanced Clinician Scientist Fellowship to M.H. (C52489/A19924) and a Cancer Research UK Cambridge Institute core grant (C9545/A29580) to M.N. M.H. and M.N. are also supported by a Medical Research Council project grant (MR/R010013/1); M.N. is also supported by the Biotechnology and Biological Sciences Research Council (BB/S013466/1).

AUTHOR CONTRIBUTIONS

Conceptualization, M.N. and M.H.; methodology, S.d.B.G., M.N., and M.H.; investigation and formal analysis, S.d.B.G., Y.I., S.G., and A.Y.; resources, S.d.B.G., K.Y., E.S., L.C., and A.Y.; software and data curation, A.C., I.O., and S.S.; writing - original draft, M.H.; writing – reviewing & editing, S.d.B.G., M.N., and M.H.; supervision, project administration, and funding acquisition, M.N. and M.H.

DECLARATION OF INTERESTS

S.d.B.G. is currently an employee of Astra Zeneca.

Received: July 2, 2020
Revised: January 6, 2021
Accepted: February 19, 2021
Published: March 16, 2021

REFERENCES

Acosta, J.C., O'Loghlen, A., Banito, A., Guijarro, M.V., Augert, A., Raguz, S., Fumagalli, M., Da Costa, M., Brown, C., Popov, N., et al. (2008). Chemokine signaling via the CXCR2 receptor reinforces senescence. Cell *133*, 1006–1018.

Bonavita, E., Bromley, C.P., Jonsson, G., Pelly, V.S., Sahoo, S., Walwyn-Brown, K., Mensurado, S., Moeini, A., Flanagan, E., Bell, C.R., et al. (2020). Antagonistic Inflammatory Phenotypes Dictate Tumor Fate and Response to Immune Checkpoint Blockade. Immunity *53*, 1215–1229.e8.

Böttcher, J.P., Bonavita, E., Chakravarty, P., Blees, H., Cabeza-Cabrerizo, M., Sammicheli, S., Rogers, N.C., Sahai, E., Zelenay, S., and Reis E Sousa, C. (2018). NK Cells Stimulate Recruitment of cDC1 into the Tumor Microenvironment Promoting Cancer Immune Control. Cell *172*, 1022–1037.e14.

Carlson, C.M., Frandsen, J.L., Kirchhof, N., McIvor, R.S., and Largaespada, D.A. (2005). Somatic integration of an oncogene-harboring Sleeping Beauty transposon models liver tumor development in the mouse. Proc. Natl. Acad. Sci. USA *102*, 17059–17064.

Chien, Y., Scuoppo, C., Wang, X., Fang, X., Balgley, B., Bolden, J.E., Premsrirut, P., Luo, W., Chicas, A., Lee, C.S., et al. (2011). Control of the senescence-associated secretory phenotype by NF-κB promotes senescence and enhances chemosensitivity. Genes Dev. *25*, 2125–2136.

Collado, M., Gil, J., Efeyan, A., Guerra, C., Schuhmacher, A.J., Barradas, M., Benguría, A., Zaballos, A., Flores, J.M., Barbacid, M., et al. (2005). Tumour biology: senescence in premalignant tumours. Nature *436*, 642.

Dagouassat, M., Gagliolo, J.-M., Chrusciel, S., Bourin, M.-C., Duprez, C., Caramelle, P., Boyer, L., Hue, S., Stern, J.-B., Validire, P., et al. (2013). The cyclooxygenase-2-prostaglandin E2 pathway maintains senescence of chronic obstructive pulmonary disease fibroblasts. Am. J. Respir. Crit. Care Med. *187*, 703–714.

Dobin, A., Davis, C.A., Schlesinger, F., Drenkow, J., Zaleski, C., Jha, S., Batut, P., Chaisson, M., and Gingeras, T.R. (2013). STAR: ultrafast universal RNA-seq aligner. Bioinformatics *29*, 15–21.

Eggert, T., Wolter, K., Ji, J., Ma, C., Yevsa, T., Klotz, S., Medina-Echeverz, J., Longerich, T., Forgues, M., Reisinger, F., et al. (2016). Distinct Functions of Senescence-Associated Immune Responses in Liver Tumor Surveillance and Tumor Progression. Cancer Cell *30*, 533–547.

Gallois, C., Habib, A., Tao, J., Moulin, S., Maclouf, J., Mallat, A., and Lotersztajn, S. (1998). Role of NF-kappaB in the antiproliferative effect of endothelin-1 and tumor necrosis factor-alpha in human hepatic stellate cells. Involvement of cyclooxygenase-2. J. Biol. Chem. *273*, 23183–23190.

Hari, P., Millar, F.R., Tarrats, N., Birch, J., Quintanilla, A., Rink, C.J., Fernández-Duran, I., Muir, M., Finch, A.J., Brunton, V.G., et al. (2019). The innate immune sensor Toll-like receptor 2 controls the senescence-associated secretory phenotype. Sci. Adv. *5*, eaaw0254.

Harrow, J., Frankish, A., Gonzalez, J.M., Tapanari, E., Diekhans, M., Kokocinski, F., Aken, B.L., Barrell, D., Zadissa, A., Searle, S., et al. (2012). GENCODE: the reference human genome annotation for The ENCODE Project. Genome Res. *22*, 1760–1774.

Hashemi Goradel, N., Najafi, M., Salehi, E., Farhood, B., and Mortezaee, K. (2019). Cyclooxygenase-2 in cancer: A review. J. Cell. Physiol. *234*, 5683–5699.

Hingorani, S.R., Petricoin, E.F., Maitra, A., Rajapakse, V., King, C., Jacobetz, M.A., Ross, S., Conrads, T.P., Veenstra, T.D., Hitt, B.A., et al. (2003). Preinvasive and invasive ductal pancreatic cancer and its early detection in the mouse. Cancer Cell *4*, 437–450.

Hoare, M., and Narita, M. (2018). The Power Behind the Throne: Senescence and the Hallmarks of Cancer. Annu. Rev. Cancer Biol. *2*, 175–194.

Hoare, M., Ito, Y., Kang, T.-W., Weekes, M.P., Matheson, N.J., Patten, D.A., Shetty, S., Parry, A.J., Menon, S., Salama, R., et al. (2016). NOTCH1 mediates a switch between two distinct secretomes during senescence. Nat. Cell Biol. *18*, 979–992.

Kabir, T.D., Leigh, R.J., Tasena, H., Mellone, M., Coletta, R.D., Parkinson, E.K., Prime, S.S., Thomas, G.J., Paterson, I.C., Zhou, D., et al. (2016). A miR-335/COX-2/PTEN axis regulates the secretory phenotype of senescent cancer-associated fibroblasts. Aging (Albany NY) *8*, 1608–1635.

Kalinski, P. (2012). Regulation of immune responses by prostaglandin E2. J. Immunol. *188*, 21–28.

Kang, T.-W., Yevsa, T., Woller, N., Hoenicke, L., Wuestefeld, T., Dauch, D., Hohmeyer, A., Gereke, M., Rudalska, R., Potapova, A., et al. (2011). Senescence surveillance of pre-malignant hepatocytes limits liver cancer development. Nature *479*, 547–551.

Kang, C., Xu, Q., Martin, T.D., Li, M.Z., Demaria, M., Aron, L., Lu, T., Yankner, B.A., Campisi, J., and Elledge, S.J. (2015). The DNA damage response induces inflammation and senescence by inhibiting autophagy of GATA4. Science *349*, aaa5612.

Kuilman, T., Michaloglou, C., Vredeveld, L.C.W., Douma, S., van Doorn, R., Desmet, C.J., Aarden, L.A., Mooi, W.J., and Peeper, D.S. (2008). Oncogene-induced senescence relayed by an interleukin-dependent inflammatory network. Cell *133*, 1019–1031.

Li, H., Handsaker, B., Wysoker, A., Fennell, T., Ruan, J., Homer, N., Marth, G., Abecasis, G., and Durbin, R. (2009). and Subgroup, 1000 Genome Project Data Processing (2009). The Sequence Alignment/Map format and SAMtools. Bioinformatics *25*, 2078–2079.

Liao, Y., Smyth, G.K., and Shi, W. (2014). featureCounts: an efficient general purpose program for assigning sequence reads to genomic features. Bioinformatics *30*, 923–930.

Livak, K.J., and Schmittgen, T.D. (2001). Analysis of relative gene expression data using real-time quantitative PCR and the 2(-Delta Delta C(T)) Method. Methods *25*, 402–408.

Loo, T.M., Kamachi, F., Watanabe, Y., Yoshimoto, S., Kanda, H., Arai, Y., Nakajima-Takagi, Y., Iwama, A., Koga, T., Sugimoto, Y., et al. (2017). Gut Microbiota Promotes Obesity-Associated Liver Cancer through PGE_2-Mediated Suppression of Antitumor Immunity. Cancer Discov. *7*, 522–538.

Lujambio, A., Akkari, L., Simon, J., Grace, D., Tschaharganeh, D.F., Bolden, J.E., Zhao, Z., Thapar, V., Joyce, J.A., Krizhanovsky, V., and Lowe, S.W. (2013). Non-cell-autonomous tumor suppression by p53. Cell *153*, 449–460.

Lun, A.T.L., Chen, Y., and Smyth, G.K. (2016). It's DE-licious: A Recipe for Differential Expression Analyses of RNA-seq Experiments Using Quasi-Likelihood Methods in edgeR. Methods Mol. Biol. *1418*, 391–416.

Lun, A.T.L., Richard, A.C., and Marioni, J.C. (2017). Testing for differential abundance in mass cytometry data. Nat. Methods *14*, 707–709.

Mootha, V.K., Lindgren, C.M., Eriksson, K.-F., Subramanian, A., Sihag, S., Lehar, J., Puigserver, P., Carlsson, E., Ridderstråle, M., Laurila, E., et al. (2003). PGC-1α-responsive genes involved in oxidative phosphorylation are coordinately downregulated in human diabetes. Nat Genet *34*, 267–273.

Na, Y.R., Jung, D., Yoon, B.R., Lee, W.W., and Seok, S.H. (2015). Endogenous prostaglandin E2 potentiates anti-inflammatory phenotype of macrophage through the CREB-C/EBP-β cascade. Eur. J. Immunol. *45*, 2661–2671.

Narita, M., Narita, M., Krizhanovsky, V., Nuñez, S., Chicas, A., Hearn, S.A., Myers, M.P., and Lowe, S.W. (2006). A novel role for high-mobility group a proteins in cellular senescence and heterochromatin formation. Cell *126*, 503–514.

Orjalo, A.V., Bhaumik, D., Gengler, B.K., Scott, G.K., and Campisi, J. (2009). Cell surface-bound IL-1alpha is an upstream regulator of the senescence-associated IL-6/IL-8 cytokine network. Proc. Natl. Acad. Sci. USA *106*, 17031–17036.

Ovadya, Y., Landsberger, T., Leins, H., Vadai, E., Gal, H., Biran, A., Yosef, R., Sagiv, A., Agrawal, A., Shapira, A., et al. (2018). Impaired immune surveillance accelerates accumulation of senescent cells and aging. Nat. Commun. *9*, 5435.

Parks, D.R., Roederer, M., and Moore, W.A. (2006). A new "Logicle" display method avoids deceptive effects of logarithmic scaling for low signals and compensated data. Cytometry A *69*, 541–551.

Parry, A.J., Hoare, M., Bihary, D., Hänsel-Hertsch, R., Smith, S., Tomimatsu, K., Mannion, E., Smith, A., D'Santos, P., Russell, I.A., et al. (2018). NOTCH-mediated non-cell autonomous regulation of chromatin structure during senescence. Nat. Commun. *9*, 1840.

Richard, A.C., Lun, A.T.L., Lau, W.W.Y., Göttgens, B., Marioni, J.C., and Griffiths, G.M. (2018). T cell cytolytic capacity is independent of initial stimulation strength. Nat. Immunol. *19*, 849–858.

Robinson, M.D., McCarthy, D.J., and Smyth, G.K. (2010). edgeR: a Bioconductor package for differential expression analysis of digital gene expression data. Bioinformatics *26*, 139–140.

Sagiv, A., Biran, A., Yon, M., Simon, J., Lowe, S.W., and Krizhanovsky, V. (2013). Granule exocytosis mediates immune surveillance of senescent cells. Oncogene *32*, 1971–1977.

Serrao, E.M., Kettunen, M.I., Rodrigues, T.B., Dzien, P., Wright, A.J., Gopinathan, A., Gallagher, F.A., Lewis, D.Y., Frese, K.K., Almeida, J., et al. (2016). MRI with hyperpolarised [1-13C]pyruvate detects advanced pancreatic preneoplasia prior to invasive disease in a mouse model. Gut *65*, 465–475.

Subramanian, A., Tamayo, P., Mootha, V.K., Mukherjee, S., Ebert, B.L., Gillette, M.A., Paulovich, A., Pomeroy, S.L., Golub, T.R., Lander, E.S., et al. (2005). Gene set enrichment analysis: A knowledge-based approach for interpreting genome-wide expression profiles. P. Natl. Acad. Sci. Usa *102*, 15545–15550.

Tasdemir, N., Banito, A., Roe, J.-S., Alonso-Curbelo, D., Camiolo, M., Tschaharganeh, D.F., Huang, C.-H., Aksoy, O., Bolden, J.E., Chen, C.-C., et al. (2016). BRD4 Connects Enhancer Remodeling to Senescence Immune Surveillance. Cancer Discov. *6*, 612–629.

Toso, A., Revandkar, A., Di Mitri, D., Guccini, I., Proietti, M., Sarti, M., Pinton, S., Zhang, J., Kalathur, M., Civenni, G., et al. (2014). Enhancing chemotherapy efficacy in Pten-deficient prostate tumors by activating the senescence-associated antitumor immunity. Cell Rep. *9*, 75–89.

van der Maaten, L. (2014). Accelerating t-SNE using Tree-Based Algorithms. J. Mach. Learn. Res. *15*, 3221–3245.

Wang, D., Patel, V.V., Ricciotti, E., Zhou, R., Levin, M.D., Gao, E., Yu, Z., Ferrari, V.A., Lu, M.M., Xu, J., et al. (2009). Cardiomyocyte cyclooxygenase-2 influences cardiac rhythm and function. Proc. Natl. Acad. Sci. USA *106*, 7548–7552.

Wickham, H. (2009). ggplot2: Elegant Graphics for Data Analysis (Springer Science & Business Media).

Zdanov, S., Bernard, D., Debacq-Chainiaux, F., Martien, S., Gosselin, K., Vercamer, C., Chelli, F., Toussaint, O., and Abbadie, C. (2007). Normal or stress-induced fibroblast senescence involves COX-2 activity. Exp. Cell Res. *313*, 3046–3056.

Zelenay, S., van der Veen, A.G., Böttcher, J.P., Snelgrove, K.J., Rogers, N., Acton, S.E., Chakravarty, P., Girotti, M.R., Marais, R., Quezada, S.A., et al. (2015). Cyclooxygenase-Dependent Tumor Growth through Evasion of Immunity. Cell *162*, 1257–1270.

STAR★METHODS

KEY RESOURCES TABLE

REAGENT or RESOURCE	SOURCE	IDENTIFIER
Antibodies		
Rabbit anti-COX2	Cell Signaling	Cat# 12282; RRID:AB_2571729
Mouse anti-HRAS	Calbiochem	#OP-23, RRID:AB_10682076
Mouse anti-IL1A	R&D Systems	MAB200, RRID:AB_2295862
Mouse anti-IL6	R&D Systems	MAB2061, RRID:AB_2127616
Mouse anti-IL8	R&D Systems	MAB208, RRID:AB_2249110
Mouse anti-β-Actin	Sigma	A5441, RRID:AB_476744
Rabbit anti-C/EBPβ	Santa Cruz	sc-150, RRID:AB_2260363
Mouse anti-IκBα	Cell Signaling	# 4814, RRID:AB_390781
Rabbit anti-Prostaglandin E2	Abcam	# ab2318, RRID:AB_302974
Mouse anti-Cyclin A2	Sigma	# C4710, RRID:AB_1078603
Mouse anti-NRAS	Santa Cruz	# sc-31, RRID:AB_628041
Mouse anti-p21	BD Biosciences	# 556431, RRID:AB_396415
Rabbit anti-ki67	Bethyl	IHC-00375, RRID:AB_1547959
Rat anti-Ly6C	Abcam	ab15627, RRID:AB_302004
Rabbit anti-CD11c	Cell Signaling	# 97585, RRID:AB_2800282
Rabbit anti-Cxcl1	Abcam	# ab86436, RRID:AB_2087574
Rat anti-Foxp3	Thermo	# 14-5773-37, RRID:AB_2865133
Rat anti-Cxcr2-BUV737	BD Biosciences	# 748680, RRID:AB_2873084
Rat anti-Ccr2-BV421	Biolegend	# 150605, RRID:AB_2571913
Rat anti-F4/80-BV711	Biolegend	# 123147, RRID:AB_2564588
Rat anti-Gr-1-APC	Biolegend	# 108411, RRID:AB_313376
Rat anti-CD45-AF700	Biolegend	# 103127, RRID:AB_493714
Hamster anti-CD11c-FITC	Biolegend	# 117305, RRID:AB_313774
Rat anti-Ly6C-PerCP-Cy5.5	Biolegend	# 128011, RRID:AB_1659242
Rat anti-CD68-PE-Cy7	Biolegend	# 137015, RRID:AB_2562947
Rat anti-CD3-PE	Biolegend	# 100205, RRID:AB_312662
Rat anti-CD19-PE	Biolegend	# 115507, RRID:AB_313642
Rat anti-CD45R/B220-PE	Biolegend	# 103207, RRID:AB_312992
Mouse anti-NK1.1-PE	Biolegend	# 108707, RRID:AB_313394
Rat anti-Gr-1-141Pr	Fluidigm	#3141005B
Hamster anti-CD11c-142Nd	Fluidigm	# 3142003B, RRID:AB_2814737
Mouse anti-MHC class 1-144Nd	Fluidigm	# 3144016B, RRID:AB_2687831
Hamster anti-CD69-Nd145	Fluidigm	#3145005B
Rat anti-F4/80-146Nd	Fluidigm	#3146008B
Rat anti-CD45-147Sm	Fluidigm	# 3147003B, RRID:AB_2811243
Rat anti-CD11b-148Nd	Fluidigm	# 3148003B, RRID:AB_2814738
Rat anti-CD19-149Sm	Fluidigm	# 3149002B, RRID:AB_2814679
Rat anti-CD25-151Eu	Fluidigm	# 3151007B, RRID:AB_2827880
Hamster anti-CD3e-152Sm	Fluidigm	# 3152004, RRID:AB_2687836
Rat anti-TER119-154Sm	Fluidigm	#3154005B
Hamster anti-TCRg/d-159Tb	Fluidigm	# 3159012B
Rat anti-B220-160Gd	Fluidigm	# 3160012B
Rat anti-Ly6C-162Dy	Fluidigm	# 3162014B

(*Continued on next page*)

Continued

REAGENT or RESOURCE	SOURCE	IDENTIFIER
Hamster anti-CD49b-164Dy	Fluidigm	# 3164011B
Rat anti-CD8a-168Er	Fluidigm	# 3168003B, RRID:AB_2811241
Hamster anti-TCRb-169Tm	Fluidigm	# 3169002B, RRID:AB_2827883
Mouse anti-NK1.1-170Er	Fluidigm	# 3170002B, RRID:AB_2885023
Rat anti-CD44-171Yb	Fluidigm	# 3171003B
Rat anti-CD4-172Yb	Fluidigm	# 3172003B, RRID:AB_2811242
Rat anti-CD127-175Lu	Fluidigm	# 3175006B
Rat anti-CD278-176Yb	Fluidigm	# 3176014B
Rat anti-CD68-FITC	Thermo	# MA5-16676, RRID:AB_2538170
Mouse anti-FITC-174Yb	Fluidigm	# 3174006B
Rat anti-CD115(CSF1R)-144Nd	Fluidigm	# 3144012B
Mouse anti-CX3CR1-164Dy	Fluidigm	# 3164023B, RRID:AB_2832247
Rat anti-CD206-169Tm	Fluidigm	# 3169021B, RRID:AB_2832249
Rat anti-CD169(Siglec1)-170Er	Fluidigm	# 3170018B, RRID:AB_2885022
Hamster anti-CD103-PE	Biolegend	# 121405, RRID:AB_535948
Mouse anti-PE-165Ho	Fluidigm	# 3165015, RRID:AB_2714168
Rat anti-Vsig4-APC	Thermo	# 17-5752-82, RRID:AB_2637429
Mouse anti-APC-176Yb	Fluidigm	# 3176007B, RRID:AB_2811236
Biological samples		
Mouse pancreas tissue from lsl-Kras^{G12D} and p48-Cre; lsl-Kras^{G12D} lines	Serrao et al., 2016	N/A
Chemicals, peptides, and recombinant proteins		
4-hydroxytamoxifen	Sigma	CAS: 68392-35-8
Etoposide	Sigma	CAS: 33419-42-0
Celecoxib	Tocris	CAS: 169590-42-5
NS398	Tocris	CAS: 123653-11-2
Prostaglandin E_2	Sigma	CAS: 363-24-6
16,16 Dimethyl-Prostaglandin E_2	Tocris	CAS: 39746-25-3
PF04418948	Sigma	CAS: 1078166-57-0
AH6809	Sigma	CAS: 33458-93-4
MK2894	MedChemExpress	CAS: 1006036-87-8
AH23848	Sigma	CAS: 81496-19-7
Liver Dissociation Kit, mouse	Miltenyi Biotec	130-105-807
Critical commercial assays		
DAKO Envision kit	Agilent	K400311-2, K400111-2
Prostaglandin E2 ELISA	R&D systems	KGE004B
Deposited data		
mRNASeq data from growing and RIS IMR90 cells treated with or without Celecoxib	This paper	GEO: GSE145650
mRNASeq data from growing, RIS and DDIS IMR90 cells	Hoare et al., 2016	GEO: GSE72407
Experimental models: cell lines		
IMR90 human diploid fibroblasts	ATCC	ATCC CCL-186; RRID:CVCL_0347
MRC-5 human diploid fibroblasts	ATCC	ATCC CCL-171; RRID:CVCL_0440
ESF human diploid fibroblasts	Jesus Gil	N/A
YT human NK cell line	DSMZ	ACC-434; RRID:CVCL_1797

(*Continued on next page*)

Continued

REAGENT or RESOURCE	SOURCE	IDENTIFIER
Experimental models: organisms/strains		
Mouse: *Ptgs2*^{tm1Gaf}	Garret Fitzgerald	MGI: 3844713
Oligonucleotides		
qPCR primers, listed in Table S2	Hoare et al., 2016 and this paper	N/A
Recombinant DNA		
pLNCX2 ER:HRAS^{G12V}	Addgene	#67844
pBabe-CEBPB-LAP*	Daniel Peeper, Kuilman et al., 2008	N/A
pBabe-IκBα-'super repressor' (S32A, S36A)	Addgene	#15291
pMSCV-miR30-shPTGS2#1	This paper	N/A
pMSCV-miR30-shPTGS2#3	This paper	N/A
pRetro-Super empty vector	Daniel Peeper, Kuilman et al., 2008	N/A
pRetro-Super shCEBPB#1	Daniel Peeper, Kuilman et al., 2008	N/A
pRetro-Super shCEBPB#4	Daniel Peeper, Kuilman et al., 2008	N/A
pWZL-N1ICD-Flag	Hoare et al., 2016	N/A
pPGK-SB13	Lars Zender, Kang et al., 2011	N/A
pCAGGS-NRAS$^{G12V / D38A}$-IRES-mVenus	Hoare et al., 2016	N/A
pCAGGS-NRAS^{G12V}-IRES-mVenus	Hoare et al., 2016	N/A
pCAGGS-NRAS$^{G12V / D38A}$-IRES >> Cre	This paper	N/A
pCAGGS-NRAS^{G12V}-IRES >> Cre	This paper	N/A
Software and algorithms		
HALO	IndicaLabs	https://indicalab.com/halo/
FlowJo	FlowJo LLC	https://www.flowjo.com/
Prism, v8	Graphpad Software	https://www.graphpad.com
STAR, v2.5.3	(Dobin et al., 2013)	https://github.com/alexdobin/STAR
FastQC, v.0.11.5	Simon Andrews, Babraham Institute	https://www.bioinformatics.babraham.ac.uk/projects/fastqc/
featureCounts, v.1.5.2	(Liao et al., 2014)	http://subread.sourceforge.net/
cydar	Lun et al., 2017	http://bioconductor.org/packages/release/bioc/html/cydar.html
Samtools	(Li et al., 2009)	http://samtools.sourceforge.net/
edgeR	(Robinson et al., 2010)	https://bioconductor.org/packages/release/bioc/html/edgeR.html
Rtsne	Van der Maaten, 2014, https://jmlr.org/papers/volume15/vandermaaten14a/vandermaaten14a.pdf	https://cran.r-project.org/web/packages/Rtsne/index.html
ggplot2	(Wickham, 2009)	https://cran.r-project.org/web/packages/ggplot2/index.html
VennDiagram, v1.6.20	R CRAN	https://cran.r-project.org/web/packages/VennDiagram/
TCseq, v1.2.0	Wu M, Gu L (2020). TCseq: Time course sequencing data analysis. R package version 1.14.0.	https://bioconductor.org/packages/release/bioc/html/TCseq.html
enrichR, v2.1	R CRAN	https://cran.r-project.org/web/packages/enrichR/
GSEA	(Subramanian et al., 2005; Mootha et al., 2003)	https://www.gsea-msigdb.org/gsea/index.jsp

RESOURCE AVAILABILITY

Lead contact

Further information and requests for resources and reagents should be directed to and will be fulfilled by the lead contact, Matthew Hoare (mwh20@cam.ac.uk).

Materials availability

This study generated plasmids with transposons containing NRAS^{G12V} and Cre-recombinase outside of the transposon for HDTV injection. These plasmids are available from the lead contact upon request with a completed Materials Transfer Agreement.

Data and code availability

The mRNA-sequencing data generated for this study have been deposited at the Gene expression omnibus (GEO) with the accession number GEO: GSE145650.

EXPERIMENTAL MODELS

In vitro cell culture

IMR90 (ATCC CCL-186; RRID:CVCL_0347), MRC-5 (ATCC CCL-171; RRID:CVCL_0440) and ESF (a kind gift from Jesus Gil) HDFs were cultured in Dulbecco's modified Eagle's medium (DMEM) / 10% fetal calf serum (FCS) in a 5% O_2 / 5% CO_2 atmosphere. The YT NK cell line (DSMZ ACC-434; RRID:CVCL_1797) was grown in DMEM / 10% FCS in a 5% CO_2 atmosphere. IMR90 / NK cell co-cultures were set-up at a cell number ratio of 1:3 and performed in DMEM / 10% FCS in a 5% CO_2 atmosphere.

Cell identity was confirmed by STR (short tandem repeats) genotyping. Cells were regularly tested for mycoplasma contamination and always found to be negative.

Mouse lines and husbandry

All animal experiments were approved by the UK legal authorities, and mice were group-housed in specified pathogen-free conditions under a 12-hour light / dark cycle in accordance with the institutional guidelines of the University of Cambridge. They had free access to water and to standard mouse chow (LabDiet, PicoLab Rodent Diet 20). All experiments were commenced when the mice were at 5 - 8 weeks of age.

Frozen sperm from *Ptgs2*$^{fl/fl}$ mice (Wang et al., 2009) (*Ptgs2*^{tm1Gaf}, MGI: 3844713, a kind gift from Garret FitzGerald) was inseminated in C57BL/6 females (Charles River) and offspring were backcrossed to homozygosity. Littermates of the same sex were randomly assigned to experimental groups

METHOD DETAILS

Compounds

The following compounds were used in cultures: 100 nM 4-hydroxytamoxifen (4OHT) (Sigma), 100 μM etoposide (Sigma), 40 μM Celecoxib (CXB) (Tocris), 10 μM NS398 (NS) (Tocris), 10 μM PGE_2 (Sigma), PF04418948 (Sigma) 1 μM, AH6809 (Sigma) 10 μM, MK2894 (MedChemExpress) 1 μM, AH23848 (Sigma) 10 μM.

Vectors

The following retroviral vectors were used: pLNCX2 ER:HRAS^{G12V} (Addgene #67844) (Parry et al., 2018); pBabe-CEBPB-LAP* (a kind gift from Daniel Peeper, NKI, Amsterdam) (Hoare et al., 2016; Kuilman et al., 2008); pBabe-IκBα-'super repressor' (S32A, S36A) (A gift from William Hahn, Addgene #15291); MSCV-puro for miR30 (Narita et al., 2006) sh1 and sh3-*PTGS2* (target sequences 5′-GCAACACTTGAGTGGCTATCA-3′ and 5′-GCATCTTCCATGATGCATTAG-3′, respectively); pRetro-Super, vector, sh1- and sh4-*CEBPB* (a kind gift from Daniel Peeper (Kuilman et al., 2008)); pWZL-N1ICD-Flag (Hoare et al., 2016).

In vivo vectors and cloning of Transposon-vectors with Cre-recombinase

The following plasmids for hydrodynamic tail-vein injection were used: pPGK-SB13 (Kang et al., 2011); pCAGGS-NRAS$^{G12V / D38A}$-IRES-mVenus; pCAGGS-NRAS^{G12V}-IRES-mVenus (Hoare et al., 2016). The pCAGGS-NRAS^{G12V}-IRES, pCAGGS-NRAS$^{G12V / D38A}$-IRES (Hoare et al., 2016; Kang et al., 2011) and pPGK-SB13 (Carlson et al., 2005) have been described previously. To generate pCAGGS-NRAS^{G12V}-IRES >> Cre and pCAGGS-NRAS$^{G12V / D38A}$-IRES >> Cre, where Cre is outside of the transposon flanking sequences (See Figure S5A), we utilized HiFi cloning: we PCR-amplified PGK and Cre separately from pPGK-Cre (a kind gift from Pedro Pérez-Mancera) using the following primers:

PGK_F 5′- CTAGAGTCGACCTGCACCGGTAGCGCCAACCGGC-3′
PGK_R 5′- TTGGGCATGGTGGCGCTGCAGGTCGAAAGGCCC-3′
Cre_F 5′- CCTGCAGCGCCACCATGCCCAAGAAGAAGAGG-3′

Cre_R 5′- GCTTGCATGCCTGCACTTTCCTCAGAAGCCATAG-3′

The obtained fragments were inserted into SbfI-linearized pCAGGS-NRAS^{G12V}-IRES and pCAGGS-NRAS$^{G12V / D38A}$-IRES vectors, before using a HiFi DNA Assembly Kit (New England Biolabs) following the manufacturer's instructions. Vectors for hydrodynamic injection were prepared with the QIAGEN EndoFree MaxiPrep kit.

NK-cell chemotaxis experiments

NK-cell chemotaxis experiments were performed and analyzed as described elsewhere (Tasdemir et al., 2016) with the following variations: NK cells were stained with the CellTracker Red CMTPX Dye (Thermo Fisher Scientific) (according to the manufacturer's instructions) and then seeded into 6-well plates containing proliferating or senescent mVenus-expressing IMR90 cells also expressing the indicated shRNAs, at 60% confluency. Cocultures were imaged over time using an Incucyte-HD or Incucyte-Zoom device (Essen Bioscience) in a 5% CO_2 atmosphere, using a 20x objective and the 488 nm and 561 nm laser excitations. Images were captured every 45 minutes, starting 30 minutes after NK-cell seeding onto the IMR90 cultures. Cell proliferation was determined through repeated-measures of confluency on phase or epifluorescent imaging.

Expression profiling by mRNA sequencing and analysis

RNA was extracted using the QIAGEN RNeasy plus kit according to manufacturer's instructions and RNA quality checked using a 4200 Tapestation Bioanalyser (Agilent). mRNA-Seq libraries were prepared from 8 biological replicates of each condition as previously described (Hoare et al., 2016), using the Illumina Truseq Stranded mRNA kit and then sequenced on an Illumina HiSeq 4000.

Sequencing alignment

mRNA-seq libraries were quality checked using the FastQC tool from the Babraham Institute. Reads were mapped to the Human reference genome hg19 with the STAR (version 2.5.0b) aligner (Harrow et al., 2012) and uniquely mapping reads were selected for further analyses. Read counts were estimated per gene using the featureCounts tool from the subread package against the gene annotation from GENCODE19, using the paired-end and the strand-specific options.

Differential expression analysis

The R package edgeR was used for pairwise differential expression analysis between each set of conditions. Significantly differentially expressed genes were selected using glmTREAT from edgeR at False Discovery Rate (FDR) 0.05.

Gene trajectories

Soft clustering (cmeans) was performed using the TCseq R package for grouping genes by their behavior across the different conditions studied using z-score scaled and TMM-normalized log-counts per million summarized across replicates. The gene sets used for clustering were genes differentially expressed in the RAS + CXB condition relative to the RAS one, as well as a secretome set of interest. The latter was determined by selecting secretome genes (Hoare et al., 2016) which were expressed in at least one of the conditions (log-counts-per-million greater than 3), resulting in a gene list of 1132 genes. These values were determined using the edgeR R package. Gene membership to a cluster was determined by selecting genes with a membership score of at least 0.5.

Venn Diagram

Venn diagrams were drawn using the VennDiagram R package using various pairs of gene sets selected from the differential expression analysis. The secretome gene list was derived from Hoare et al. (2016).

Enrichment

Gene enrichment analysis was performed using EnrichR via the R package enrichR against the TRRUST Transcription Factors databases (2019 versions).

BrdU incorporation, colony formation and SA-β-galactosidase assays

Cellular proliferation by BrdU incorporation, colony formation and SA-β-galactosidase analyses have been described previously (Narita et al., 2006).

Laser scanning cytometry

Cell counting was performed using Laser scanning cytometry on an iCys Research Imaging Cytometer (CompuCyte, Cambridge, MA) using anti-IL8 and anti-COX2 antibodies, appropriate fluorescent-tagged secondary antibodies (as below) and counter-staining with DAPI.

mRNA expression by quantitative RT-PCR

RNA was extracted using the QIAGEN RNeasy plus kit and reverse transcribed to cDNA using the high-capacity reverse transcription kit (Applied Biosystems). qRT-PCR was performed as described before (Narita et al., 2006) with relative expression determined by

the $2^{-\Delta\Delta Ct}$ method (Livak and Schmittgen, 2001) using β-Actin (*ACTB*) as an internal control. Primer sequences are detailed in Table S2.

Protein expression by immunoblotting and immunofluorescence

Immunofluorescence and immunoblotting, on SDS-PAGE gels were performed as reported previously (Hoare et al., 2016). The following antibodies were used in this study: anti-COX2 (Cell Signaling, 12282, 1:1000); anti-HRAS (Calbiochem, OP-23, 1:500); anti-IL1a (R&D systems, MAB200, 1:100); anti-IL6 (R&D systems, MAB2061, 1:250); anti-IL8 (R&D systems, MAB208, 1:500); anti-β-Actin (Sigma, A5441, 1:5000); anti-C/EBPβ (Santa-Cruz, sc-150, 1:500); anti-IkBα (Cell Signaling, 4814, 1:1000); anti-PGE_2 (Abcam, ab2318, 1:100); anti-CyclinA2 (Sigma, C4710, 1:500).

Prostaglandin E2 ELISA

Conditioned media was harvested from 1.0×10^5 IMR90 cells cultured in serum-free media for 16 hours, before filtration through a 0.22 μm filter and then centrifuged at 4000 g for 20 minutes. PGE_2 detection was performed according to the manufacturer's instructions (Prostaglandin E2 Parameter Assay, R&D systems, KGE004B). The PGE_2 concentration of the samples was inferred from the standard curve generated using known concentrations of PGE_2.

Mouse maintenance and experiments

Ptgs2$^{fl/fl}$ mice underwent hydrodynamic tail vein injection as previously described at 5 – 8 weeks of age (Kang et al., 2011); briefly, 20 μg of appropriate vector and 5 μg of SB13 transposase-containing plasmid were diluted in sterile-filtered normal saline to a total volume of 10% of the body weight of the animal, before being injected into the lateral tail vein in under 10 s.

To replace PGE_2 *in vivo* 16,16-Dimethyl Prostaglandin E2 ($dmPGE_2$) (Tocris, 4027) 200 μg/kg in 100 μL sunflower oil was injected into the peritoneum (IP) every 24 hours on days 5 to 8 after HDTV injection. To analyze hepatocyte proliferation mice were IP injected with EdU (100 μL of 10mg/μl stock solution) on D5 post-HDTV injection before culling on D6.

Pancreas tissue was obtained from LSL-Kras^{G12D}; p48-Cre (KC) mice and aged-matched LSL-Kras^{G12D} control mice as described previously (Serrao et al., 2016).

Immunohistochemistry

Immunohistochemistry was performed as reported previously (Hoare et al., 2016). Formalin fixed paraffin-embedded mouse tissues were stained with the following antibodies: anti-Cox2 (as above); anti-NRAS (Santa Cruz, sc-31,1:100); anti-p21 (BD, 556431, 1:50); anti-ki67 (Bethyl, IHC-00375, 1:1000); anti-Ly6C (Abcam, ab15627, 1:400); anti-Cd11c (Cell Signaling, 97585, 1:350); anti-Cxcl1 (Abcam, ab86436, 1:100); anti-PGE_2 (Abcam, ab2318, 1:100); anti-Foxp3 (eBioscience, 14-5773, 1:100); after proteinase K digestion (Ly6C) or heat-induced epitope retrieval in citrate (pH6) or Tris-EDTA (pH9) buffers before visualization using the DAKO Envision kit according to manufacturer's instructions and counterstaining with hematoxylin. For fluorescent labeling we utilized appropriate fluorochrome-tagged secondary antibodies (Life Technologies). For EdU staining (ThermoFisher C10638), the same protocol was followed, with the following extra step: after antigen retrieval, 3% BSA washes in PBS were performed twice, and CliCK-iT reaction cocktail was added for 30 min following the manufacturer's instructions.

All slides were scanned on a Leica AT2 at 20x magnification and a resolution of 0.5 μm/pixel. Following digitization, image analysis was performed as described previously using HALO (Indicalabs) (Hoare et al., 2016).

Mass-cytometry and data analysis

Transduced murine livers were dissociated using the mouse liver dissociation kit (Miltenyi Biotec) and submitted to multiplexed mass-cytometry based deep immunophenotyping, according to the manufacturer's instructions (Fluidigm). Immune cells were stained with 5 mL cisplatin in PBS at room temperature for 5 min followed by washing with Maxpar staining buffer (MSB). Resuspended cells were fixed by FixI buffer at room temperature for 10 min. Cell suspensions were washed four times in MSB and stored at −80°C as cell pellets in 1 million cell aliquots. Cell pellets were thawed on ice on day of use. Each cell pellet was resuspended in Maxpar Barcode Perm Buffer and barcoded using the palladium isotope barcodes at room temperature for 30 min. Each barcoded sample was washed twice with MSB before combining into one single cell suspension before staining with Fc-Block (BD Bioscience), followed by staining with a mixture of metal-conjugated antibodies directed against extracellular antigens at room temperature for 30 min. In some cases, fluorophore-conjugated antibodies were used as primary antibody before recognition by a metal-conjugated anti-fluorophore secondary antibody. Antibodies (Fluidigm, except if another manufacturer is specified) are detailed in Table S2 and the key resources table.

After staining, the cell pellet was washed with MSB and then incubated in Cell-ID Intercalator overnight at 4°C. The cell pellet was washed four times with MSB and submitted for data acquisition on Helios CyTOF (Fluidigm). Following acquisition, times series were normalized to internal bead standards, concatenated and de-barcoded using inbuilt software (Fluidigm).

De-barcoded mass cytometry data was analyzed using the cydar Bioconductor package (Lun et al., 2017), using a workflow similar to previously described (Richard et al., 2018). Cells were pooled across samples, and mass-labeled marker values were transformed using the Logicle transformation (Parks et al., 2006). The following filters were then imposed sequentially: Removal of calibration beads, inclusion of singlet cells using Ir191 and Ir193 DNA markers, removal of dead cells expressing high levels of a live-dead

marker, removal of non-immune cells expressing low levels of CD45, and removal of erythrocytes expressing high levels of TER119. The gates set for each of the above filters were kept constant across all samples, and the channels used for this gating step were removed from subsequent analysis.

Cells were then assigned to hyperspheres with a radius defined from a per-marker log-intensity tolerance of 0.5, and down-sampled by sampling hyperspheres at a frequency of 5-20 cells, depending on the number of cells acquired in each dataset, with 200-300 thousand hyperspheres used for subsequent differential abundance analysis. Differential abundance was analyzed using the edgeR Bioconductor package with a quasi-likelihood GLM fit (Lun et al., 2016). Significant hyperspheres were identified by analysis of deviance to detect those differentially abundant between any pair of conditions, controlling the spatial FDR at 5%. tSNE projection values were generated using the Rtsne package (van der Maaten, 2014) with a perplexity value of 50, and subsequent plots were generated with the Rtsne and ggplot2 packages. Antibodies (Fluidigm, except if another manufacturer is specified) are detailed in Table S2.

Flow-cytometry and data analysis

Intrahepatic immune cells were prepared as above and then run on a BD Fortessa flow cytometer (Becton Dickinson); antibodies used are detailed in the key resources table; data was analyzed using FlowJo V10.

TCGA analysis

Immune TCGA analysis was conducted in the R statistical computing language (http://www.R-project.org/) using ggplot2. Correlations between gene expression levels were computed using the Spearman's rank correlation test and p values were FDR corrected. Gene cluster signature expression was calculated using the geometric mean expression of all included genes. TCGA gene expression (hg19 version) and clinical data were obtained from the GDC portal (https://portal.gdc.cancer.gov/legacy-archive), RNA-sequencing data was pre-calculated continuous count data derived from the Broad analysis pipeline (full details:
https://docs.gdc.cancer.gov/Data/Bioinformatics_Pipelines/Expression_mRNA_Pipeline/).

QUANTIFICATION AND STATISTICAL ANALYSIS

Statistical analyses were conducted using GraphPad Prism 8 and R statistical software, except where indicated. Statistical details of the experiments can be found in the relevant figure legend, including the statistical tests used and the number of biological replicates. Unless otherwise stated, data are represented by the mean ± SEM. *n* values represent the number of independent experiments performed or the number of individual mice per condition. One-way ANOVA with Tukey's or Sidak's correction for multiple comparisons was used for datasets with more than 2 conditions. Student's t tests were used for two-condition comparisons. The statistical tests were justified as appropriate based on the number of samples compared and the assumed variance within populations. A p-value < 0.05 was used to indicate statistical significance.

Cell Reports, Volume *34*

Supplemental information

COX2 regulates senescence secretome composition and senescence surveillance through PGE_2

Susana Gonçalves, Kelvin Yin, Yoko Ito, Adelyne Chan, Ioana Olan, Sarah Gough, Liam Cassidy, Eva Serrao, Stephen Smith, Andrew Young, Masashi Narita, and Matthew Hoare

Supplementary Figure 1

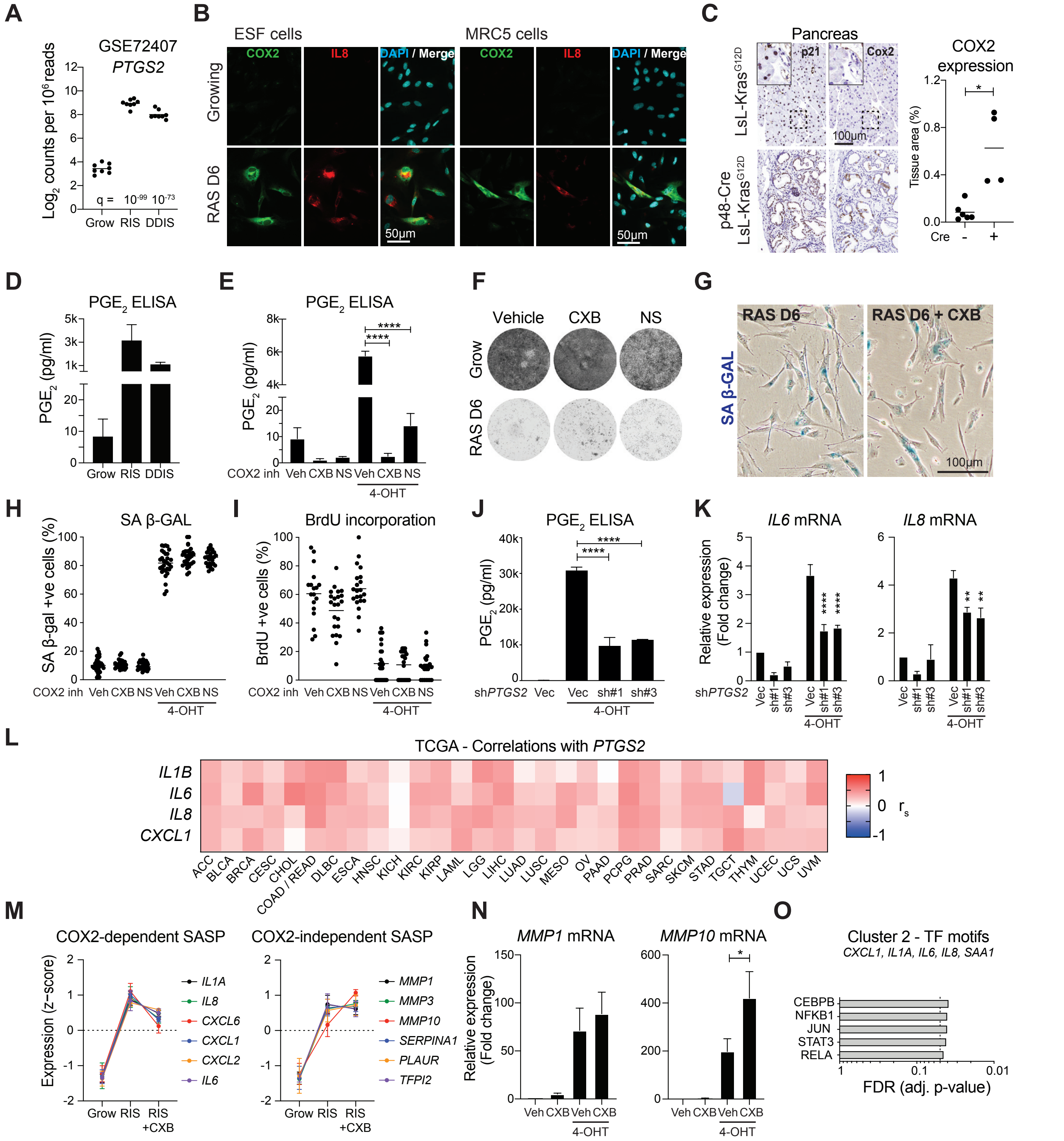

Supplementary figure 1. COX2 is upregulated in senescence and regulates SASP composition, related to Figure 1. (A) Expression of *PTGS2*, the gene that encodes COX2, from previously generated mRNA-Seq (GSE72407) data in growing, RAS-induced senescent (RIS) and DNA-damage-induced senescent (DDIS) ER:HRAS^{G12V} IMR90 cells. (B) Representative immunofluorescence of COX2 and IL8 in growing or RIS ER:HRAS^{G12V} ESF (left) or ER:HRAS^{G12V} MRC5 (right) HDFs cells (scale bar 50µm). (C) Left: representative photomicrographs of immunohistochemistry for p21 (left) and COX2 (right) of pancreatic tissue from p48-cre; LSL-Kras^{G12D} (n = 4) and matched control mice (n = 6); insets demonstrate the staining patterns within normal pancreatic ducts in the control mice (upper) and pancreatic intraepithelial neoplasia (PanIN) in the p48-cre; LSL-Kras^{G12D} mice (lower). Scale bar 100 µm. Right: quantification of Cox2-positive tissue area; bar represents the mean; * P < 0.05; statistical analysis by unpaired t-test with Welch's correction. (D) Prostaglandin E2 (PGE$_2$) levels by ELISA in growing, RIS or DDIS ER:HRAS^{G12V} IMR90 cells (n = 3 biologically independent experiments). (E) Prostaglandin E2 (PGE$_2$) levels by ELISA in growing or D6 RIS ER:HRAS^{G12V} IMR90 cells treated with vehicle (Veh), Celecoxib (40µM, CXB) or NS398 (10µM, NS); n = 3 biologically independent experiments; values are mean ± SEM; **** P ≤ 0.0001; statistical analysis by 1-way ANOVA with Sidak's multiple comparisons test. (F) Growing or RIS ER:HRAS^{G12V} IMR90 cells treated with vehicle (Veh), Celecoxib (40µM, CXB) or NS398 (10µM, NS) were analysed for colony forming capacity. (G, H & I) Growing or RIS ER:HRAS^{G12V} IMR90 cells treated with vehicle, CXB or NS were analysed for SA-β GAL expression (G, H) or BrdU incorporation (I) at D6 RAS. Example photomicrographs in (G) from indicated conditions (scale bar 100µm) and summary data in (H); n ≥ 18 biologically independent experiments; bar represents the mean. (J) PGE$_2$ levels by ELISA in growing or D6 RIS ER:HRAS^{G12V} IMR90 cells expressing shRNAs against *PTGS2* or vector control; n = 3 biologically independent experiments; values are mean ± SEM; **** P ≤ 0.0001; statistical analysis by 1-way ANOVA with Sidak's multiple comparisons test. (K) Growing or RIS ER:HRAS^{G12V} IMR90 cells, expressing shRNAs against *PTGS2* or vector control, were analysed for expression of indicated genes by qRT-PCR; n ≥ 3 biologically independent samples per condition; values are mean ± SEM; **** P ≤ 0.0001 versus RIS / vector condition; statistical analysis by 2-way ANOVA with Tukey's multiple comparisons test; values are mean ± SEM; ** P ≤ 0.01, ****P ≤ 0.0001. (L) Heatmap of correlation coefficients between expression of *PTGS2* and selected cytokines in a pan-cancer analysis of TCGA transcriptomic data; statistical analysis by Spearman's rank correlation (rs). (M) z-score normalised expression from mRNA-Seq of selected canonical SASP components, demonstrating COX2 dependent (left) and COX2-independent (right) genes. (N) Expression of *MMP1* and *MMP10* in growing or RIS ER:HRAS^{G12V} IMR90 cells treated with vehicle or CXB was analysed by qRT-PCR (n = 5 biologically independent replicates for all conditions; values are mean ± SEM; * P ≤ 0.05; statistical analysis by 1-way ANOVA with Sidak's multiple comparisons test. (O) Transcription factor motif enrichment analysis from cluster 2 genes, using TRRUST v2 database.

Supplementary figure 2

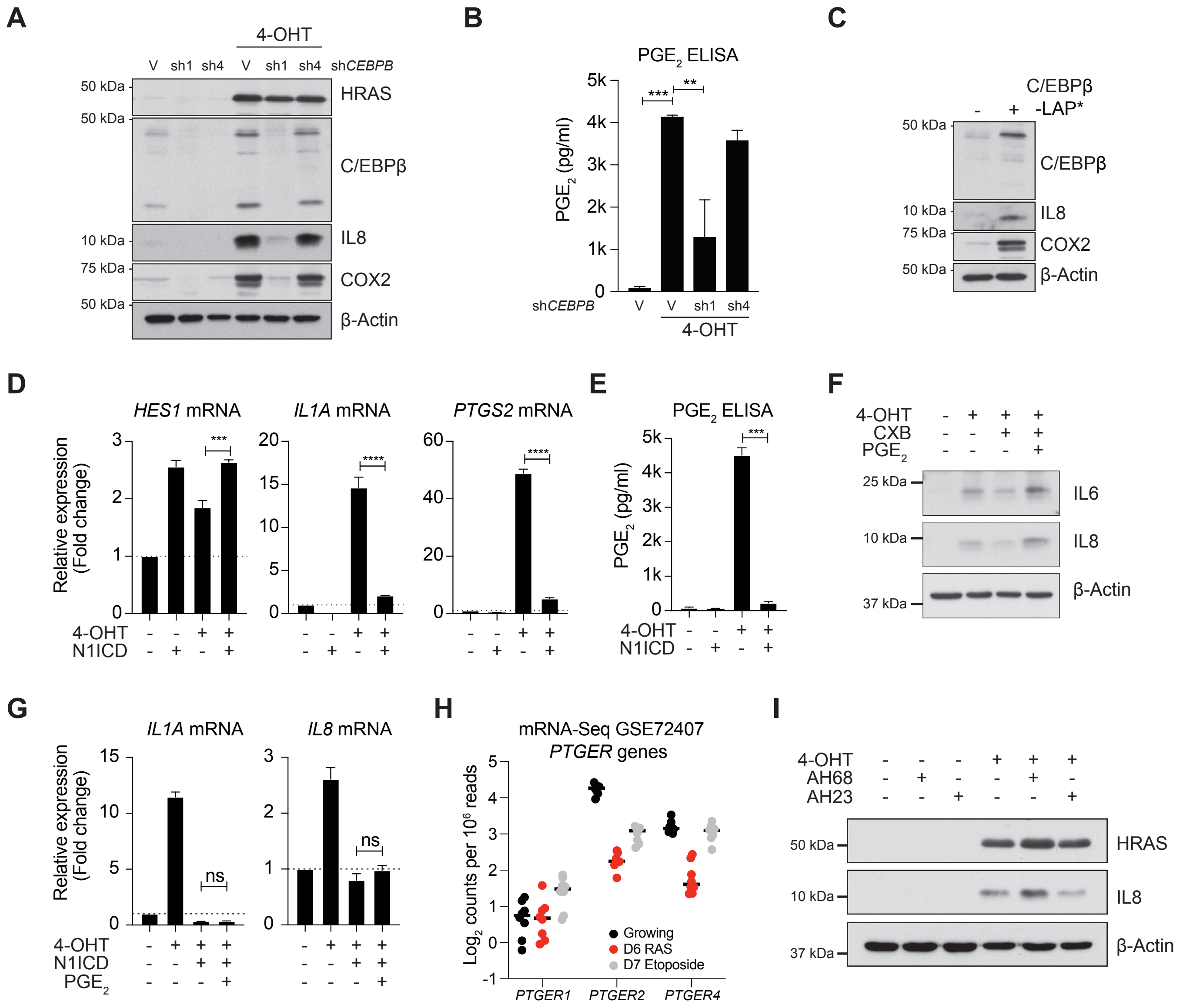

Supplementary figure 2. Upstream regulation of COX2 and downstream regulation of the SASP through PGE$_2$, related to Figure 2. (A) Growing or RIS ER:HRAS^{G12V} IMR90 cells, expressing shRNAs against *CEBPB* or vector control, were analysed for expression of indicated proteins by immunoblotting (A) and Prostaglandin E2 (PGE$_2$) levels by ELISA (B); n = 3 biologically independent experiments; values are mean ± SEM; ** $P \leq 0.01$, *** $P \leq 0.001$; statistical analysis by 1-way ANOVA with Sidak's multiple comparisons test. (C) IMR90 cells expressing the transcriptionally active LAP* form of C/EBPβ or matched vector control were analysed for the indicated proteins by immunoblotting. (D) Expression of *HES1, IL1A* and *COX2* in growing or RIS ER:HRAS^{G12V} IMR90 cells transfected with a transcriptional active NOTCH1-ICD (N1ICD) or vector control were analysed by qRT-PCR; (E) expression of PGE$_2$ was analysed in the same cells by ELISA; n = 3 biologically independent replicates for all conditions; values are mean ± SEM; statistical analysis by 1-way ANOVA with Sidak's multiple comparisons test; *** $P \leq 0.001$, **** $P \leq 0.0001$. (F) Expression of IL6 and IL8 in growing or RIS ER:HRAS^{G12V} IMR90 cells treated with vehicle or CXB and vehicle or PGE$_2$ (10µM) by immunoblotting. (G) Expression of *IL1A* and *IL8* in growing or RIS ER:HRAS^{G12V} IMR90 cells, expressing N1ICD or vector control and treated with vehicle or PGE$_2$ were analysed by qRT-PCR; n = 3 biologically independent replicates for all conditions; values are mean ± SEM; statistical analysis by 1-way ANOVA with Sidak's multiple comparisons test. (H) Expression of *PTGER* genes, encoding the EP receptors, from previously generated mRNA-Seq (GSE72407) data in growing, RAS-induced senescent (RIS) and DNA-damage-induced senescent (DDIS) ER:HRAS^{G12V} IMR90 cells. *PTGER3* is not detectable in IMR90 cells. (I) Growing or RIS ER:HRAS^{G12V} IMR90 cells, treated with vehicle, AH6809 (10µM) or AH23848 (10µM) were analysed for expression of indicated proteins by immunoblotting.

Supplementary figure 3

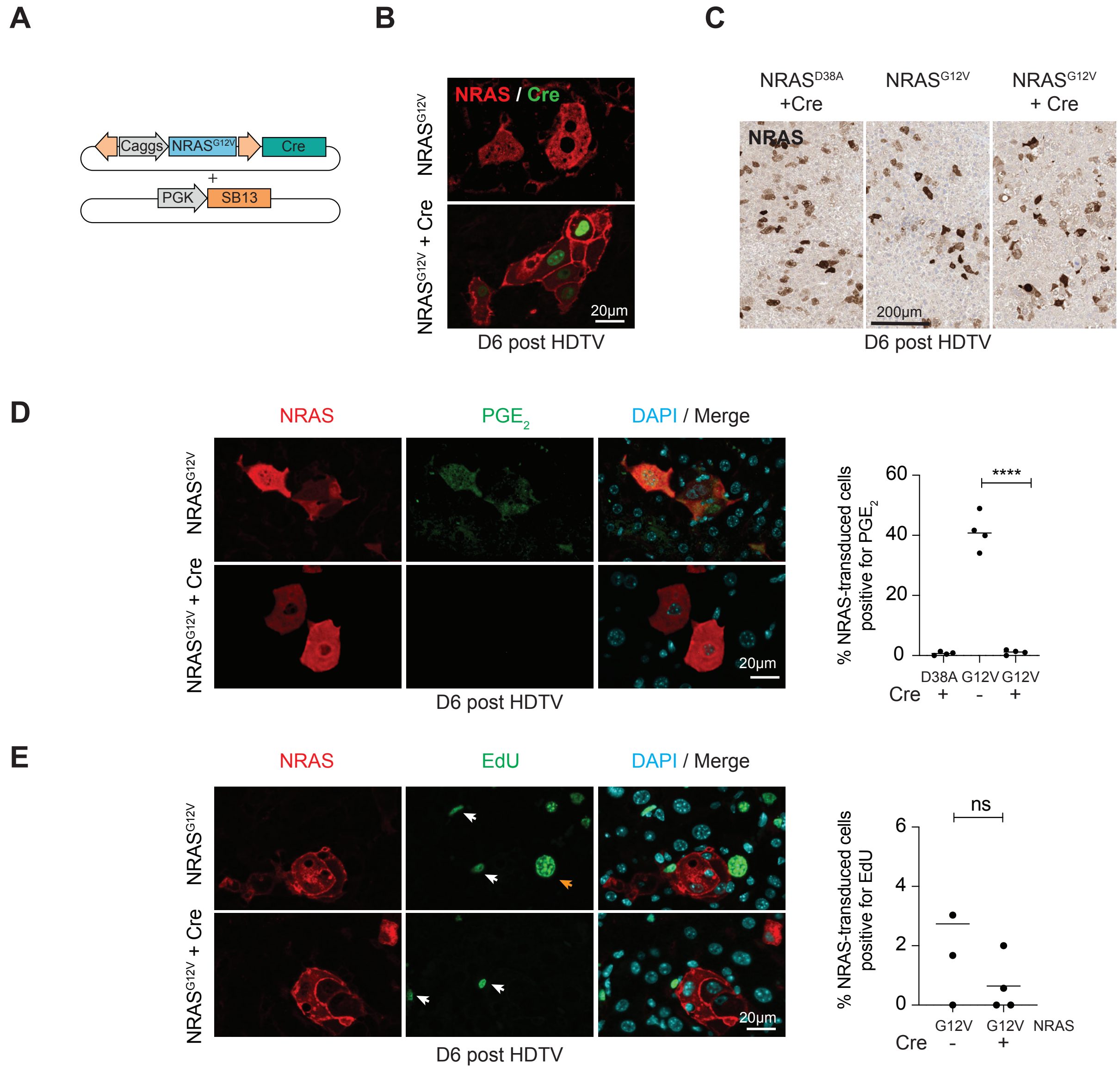

Supplementary figure 3. Cox2 regulates senescence surveillance and tumour suppression *in vivo*, related to Figure 3. (A) Cartoon schematic of the transposon and transposase-containing vectors that were used in the hydrodynamic tail-vein (HDTV) injection experiments. The Cre-recombinase is outside the transposon inverted repeat elements and so is only expressed episomally and transiently after injection. (B) Representative immunofluorescence images of NRAS and Cre in mouse livers at D6 post HDTV injection of the indicated constructs; scale bar 20µm (C) Illustrative photomicrographs of NRAS immunohistochemistry in indicated conditions at D6 post HDTV injection; scale bar 200µm. (D) Representative immunofluorescence images (left) of NRAS and PGE_2 in mouse livers at D6 post HDTV injection of the indicated constructs; scale bar 20µm and quantification of the number of NRAS+ hepatocytes that were also positive for PGE_2 staining (right) in the indicated conditions; dots are individual mice; bars are means; data analysed by 1-way ANOVA with Sidak's multiple comparisons test; ****$P \leq 0.0001$. (E) Representative immunofluorescence images (left) of NRAS and EdU in mouse livers at D6 post HDTV injection of the indicated constructs; note the EdU incorporation in immune cells (white arrowheads) and non-transduced hepatocyte (orange arrowhead); scale bar 20µm; quantification of the number of NRAS+ hepatocytes that were also positive for EdU staining (right) in the indicated conditions; dots are individual mice; bars are means; data analysed by unpaired student's t-test.

Supplementary figure 4

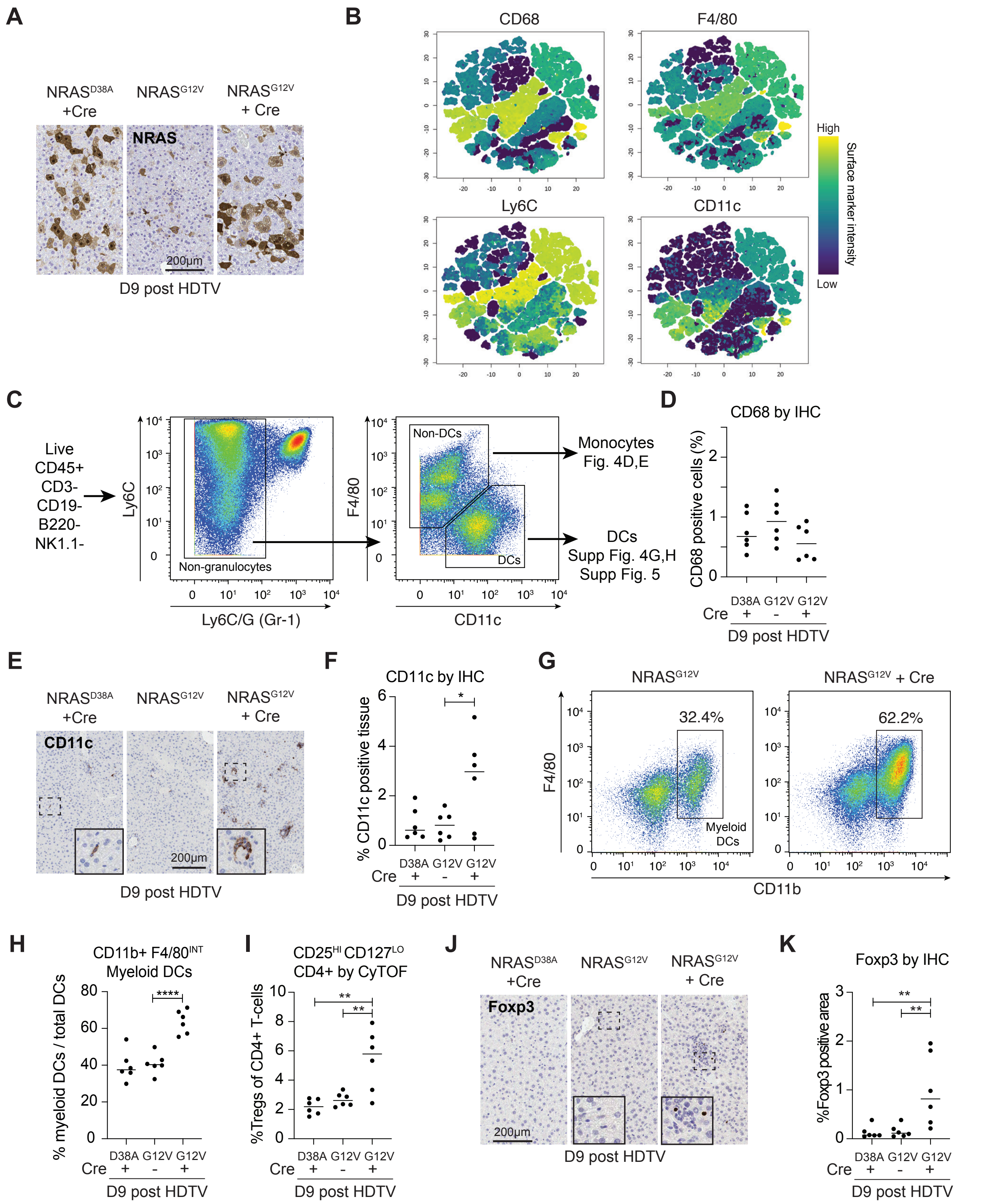

Supplementary figure 4. Cox2 controls the intrahepatic immune microenvironment during senescence surveillance, related to Figure 4. (A) Illustrative photomicrographs of NRAS IHC in indicated conditions at D9 post HDTV injection; scale bar 200µm. (B) t-SNE plots showing hyperspheres of immune cell phenotypes (see Fig. 4 B&C), coloured by median intensity of the indicated markers. The colour range is bounded by the 1st and 99th percentiles of the intensities across all cells. (C) Gating strategy to study a non-granulocytic / non-dendritic cell (DC) myeloid population in figures 4 D&E. (D) Expression of CD68 in the liver by IHC in same mice used for mass-cytometry; dots are individual mice; bars are means. (E) Illustrative photomicrographs of CD11c IHC in indicated conditions at D9 post HDTV injection of the indicated constructs; scale bar 200µm. Insets are higher magnification views of areas in the dotted dashed boxes showing CD11c+ cells. (F) Quantification of CD11c-positive area per liver section, by IHC. Dots are individual mice; bars are means; data analysed by 1-way ANOVA with Sidak's multiple comparisons test; * $P \leq 0.05$. (G, H) Live lineage-negative, dendritic cells (DC) were gated as in supplementary fig. 4C, before analysis of different DC populations based on CD11b and F4/80 expression, identifying myeloid DCs as CD11b+ and F4/80^{INT}; example dot-plots of DC populations from NRAS^{G12V} and NRAS^{G12V}+Cre injected mice are shown in (G) with quantification in (H); dots are individual mice; bars are means; data analysed by 1-way ANOVA with Sidak's multiple comparisons test; **** $P \leq 0.0001$. (I) Relative abundance of CD4+ Tregs across the indicated conditions at D9 post HDTV injection by mass cytometry. Tregs were defined by the surface expression profile CD3+CD4+CD25^{HI}CD127^{LO} and expressed as a proportion of total CD3+CD4+ cells; dots are individual mice; bars are means; data analysed by 1-way ANOVA with Sidak's multiple comparisons test; ** $P \leq 0.01$. (J) Illustrative photomicrographs of Foxp3 IHC in indicated conditions at D9 post HDTV injection of the indicated constructs; scale bar 200µm. Insets are higher magnification views of areas in the dotted dashed boxes showing Foxp3+ cells. (K) Quantification of Foxp3-positive area per liver section, by IHC. Dots are individual mice; bars are means; data analysed by 1-way ANOVA with Sidak's multiple comparisons test; ** $P \leq 0.01$.

Supplementary figure 5

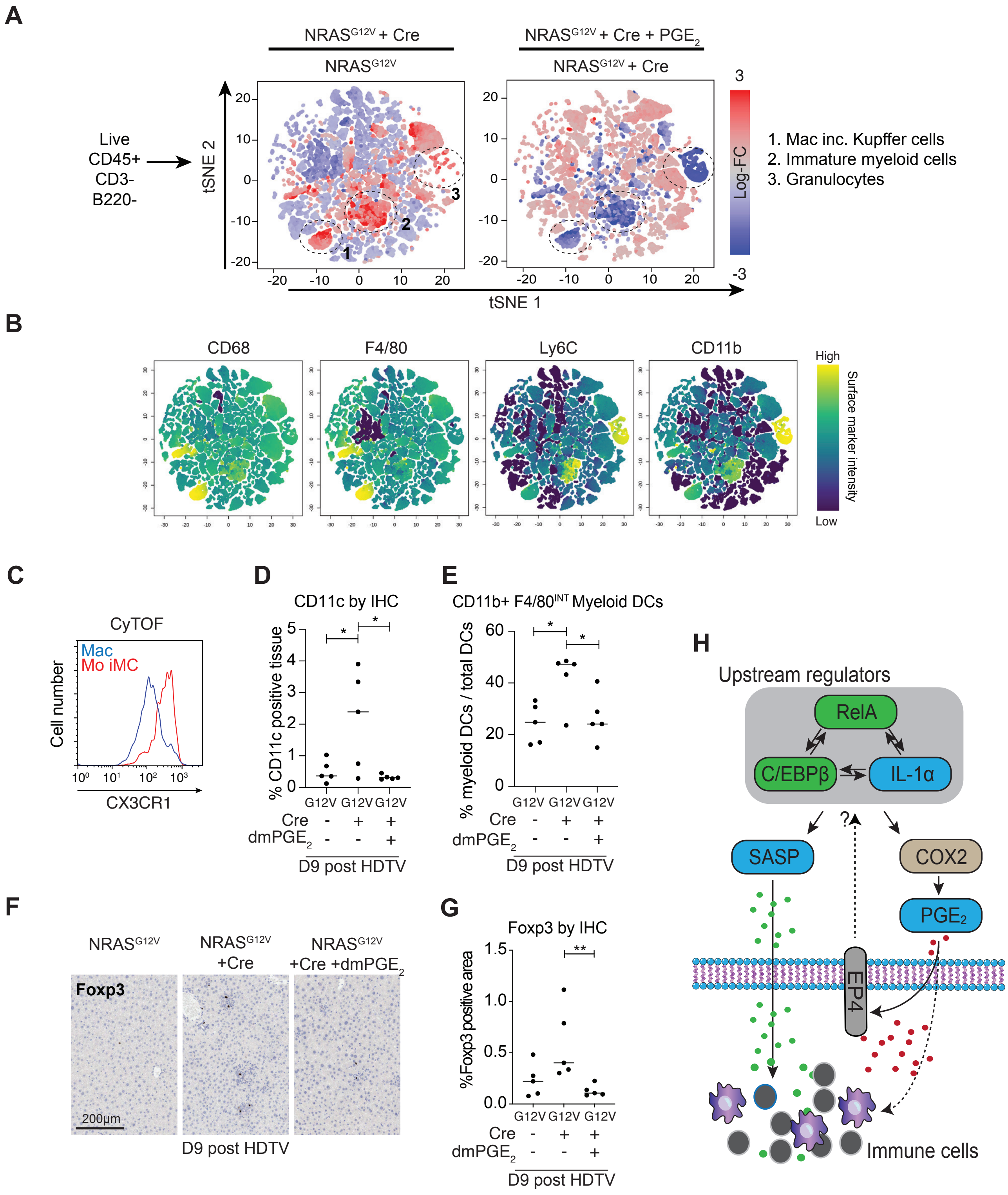

Supplementary figure 5. PGE$_2$ partially rescues Cox2-dependent immune-mediated senescence surveillance, related to Figure 5. (A) t-SNE plots of multiplexed intrahepatic immune cell mass-cytometry data from 15 mice (same conditions as Fig. 5 A-C, n = 5 per condition), using 16 metal-tagged antibodies against immunocyte surface markers (see supplementary table 2). t-SNE plots demonstrate differential abundance of CD45+, non-lymphoid cells from NRAS^{G12V} vs NRAS^{G12V}+Cre (left) and NRAS^{G12V}+Cre vs NRAS^{G12V}+Cre+dmPGE$_2$ (right) mice. Hyperspheres of immune cell phenotypes were identified by typical surface marker expression and coloured according to log-fold change in abundance between conditions. (B) t-SNE plots showing hyperspheres of immune cell phenotypes, coloured by median intensity of the indicated markers. The colour range is bounded by the 1st and 99th percentiles of the intensities across all cells. (C) Histogram demonstrating expression of CX3CR1 on iMCs (CD68^{Intermediate} / F4/80^{Low}) and macrophages (CD68^{High} / F4/80^{High}) from NRAS^{G12V}+Cre injected mouse liver. (D) Quantification of CD11c-positive area per liver section, by IHC in the indicated conditions. Dots are individual mice; bars are means; data analysed by 1-way ANOVA with Sidak's multiple comparisons test; * P ≤ 0.05. (E) Relative abundance of myeloid DCs across the indicated conditions at D9 post HDTV injection by mass cytometry; see Supp fig 4c for gating strategy. Myeloid DCs were defined by the surface expression profile CD11b+F4/80^{INT} and expressed as a proportion of total DCs; dots are individual mice; bars are means; data analysed by 1-way ANOVA with Sidak's multiple comparisons test; * P ≤ 0.05. (F) Illustrative photomicrographs of Foxp3 IHC in indicated conditions at D9 post HDTV injection of the indicated constructs; scale bar 200µm. (G) Quantification of Foxp3-positive area per liver section, by IHC. Dots are individual mice; bars are means; data analysed by 1-way ANOVA with Sidak's multiple comparisons test; ** P ≤ 0.01. (H) Putative model of COX2-dependent regulation of SASP composition and functionality. COX2 expression in RIS is C/EBPβ and NF-κB-dependent; amongst downstream products PGE$_2$ is able to bind EP4 receptors and modulate SASP expression or directly modulate immune cell functionality. Dotted lines represent indirect or uncertain pathways of regulation.
